# Supplementary material for: Tyrosine phosphorylation of CARM1 promotes its enzymatic activity and alters its target specificity
Source: Nat Commun. 2024 Apr 22;15:3415. doi: 10.1038/s41467-024-47689-4 (PMC11035800; doi:10.1038/s41467-024-47689-4)
Supplement: Supplementary file 1 — Supplementary information [file 41467_2024_47689_MOESM1_ESM.pdf]

Supplementary information

Tyrosine phosphorylation of CARM1 promotes its enzymatic activity and alters its target specificity

Hidehiro Itonaga, Adnan K. Mookhtiar, Sarah M. Greenblatt, Fan Liu, Concepcion Martinez, Daniel Bilbao, Masai Rains, Pierre-Jacques Hamard, Jun Sun, Afoma C. Umeano, Stephanie Duffort, Chuan Chen, Na Man, Gloria Mas, Luca Tottone, Tulasigeri Totiger, Terrence Bradley, Justin Taylor, Stephan Schürer, and Stephen D. Nimer

1. Supplementary Figure

2. Supplementary Tables (Key resource table)

3. Supplementary reference

1. Supplementary Figure

Supplementary Figure 1.

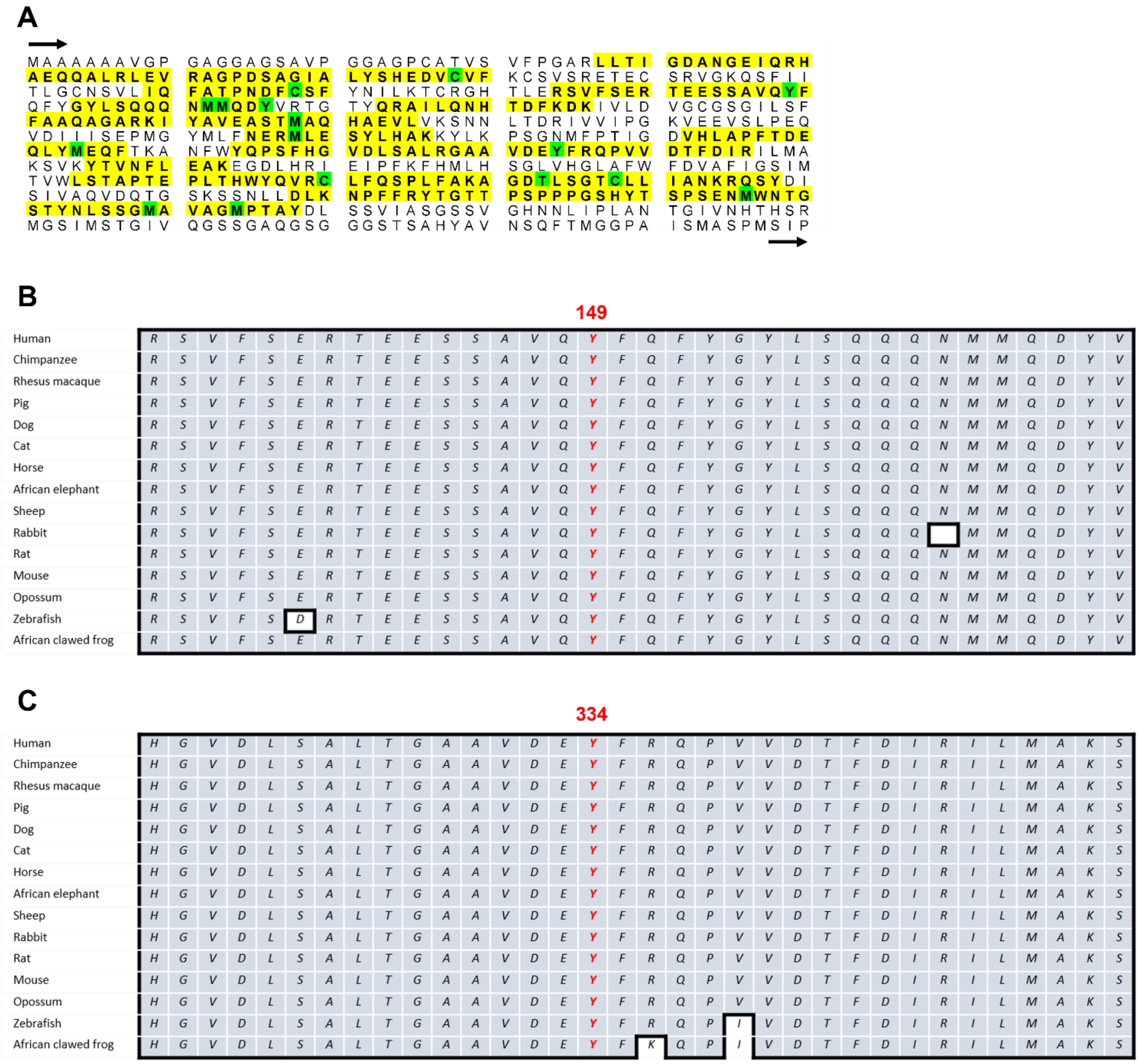

Supplementary Figure 1. Primary sequence alignment of CARM1 protein.

(A) The coverage rate of mass spectrometry analysis is 48% of full-length human CARM1 protein (indicating yellow or green marked residues), in which we checked 16 out of 20 tyrosine residues. Sequence alignment around tyrosine-149 (Y149) (B) and tyrosine-334 (Y334) (C) in CARM1. These alignments shown are evolutionally conserved, from African clawed frog to human.

Supplementary Figure 2.

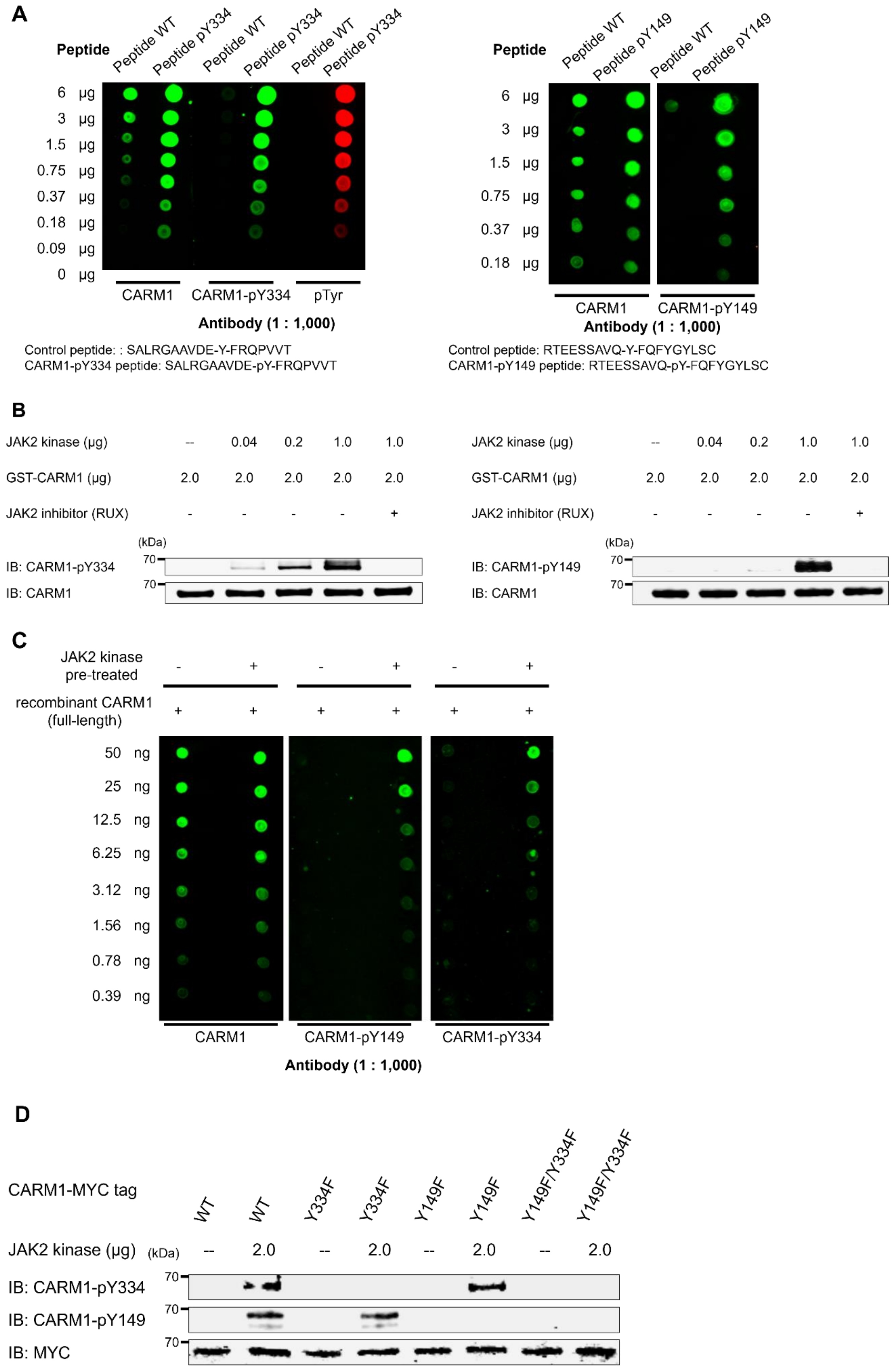

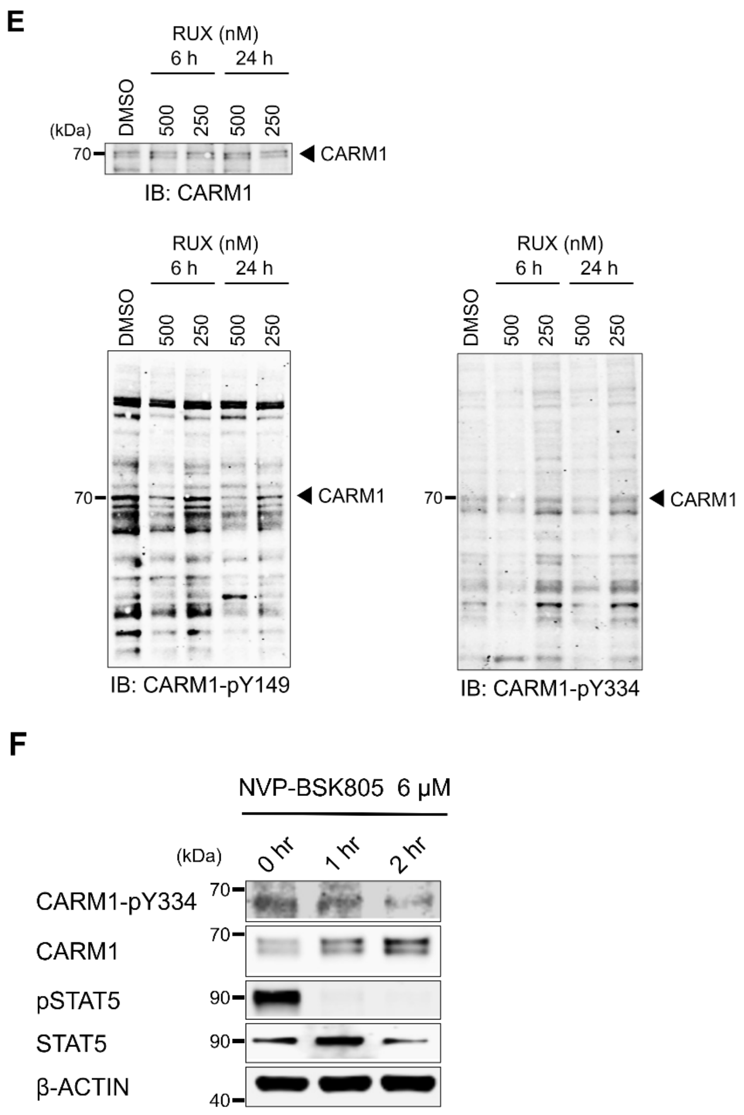

Supplementary Figure 2. Anti-CARM1phosphospecific antibodies recognize phosphorylated CARM1

(A) Dot-blot analysis showed that anti-CARM1-Y334 (left) and -Y149 (right) phosphospecific antibodies recognized phosphorylated peptides in a dose-dependent manner.

(B) Anti-CARM1-Y334 phosphospecific antibody recognized CARM1 in the presence of active JAK2 kinase in a dose dependent manner (left). Anti-CARM1-Y149 phosphospecific antibody recognized CARM1 in the presence of active JAK2 kinase in a dose dependent manner (right). In both assays, the complete abolishment of CARM1 phosphorylation by JAK1/JAK2 inhibitor (ruxolitinib, RUX; 500 nM) was observed.

(C) Dot-blot analysis showed that anti-CARM1-Y149 (middle) and -Y334 (right) phosphospecific antibodies recognized phosphorylated CARM1 proteins (full-length), generated by incubation with active JAK2 kinase.

(D) MYC-tagged CARM1 wild-type (WT), Y149F or Y334F single mutations, or Y149F/Y334F

double mutations were expressed in K562 cells. Proteins were precipitated by anti-MYC immunoprecipitation, and immunoblotted using anti-CARM1-phosphospecific antibodies following the incubation with or without recombinant active JAK2 kinase.

(E) Endogenous phosphorylated CARM1 was immunoblotted from total cell lysates of HEL cells treated with DMSO or RUX, using anti-CARM1-Y149 (left) and -Y334 (right) phosphospecific antibodies. The reduction of CARM1 phosphorylation was observed at the higher RUX concentration (500 nM).

(F) Phosphorylation of Y334 of CARM1 in HEL cells is reduced following treatment with the highly selective JAK2 inhibitor (NVP-BSK805, 6 μM).

Supplementary Figure 3.

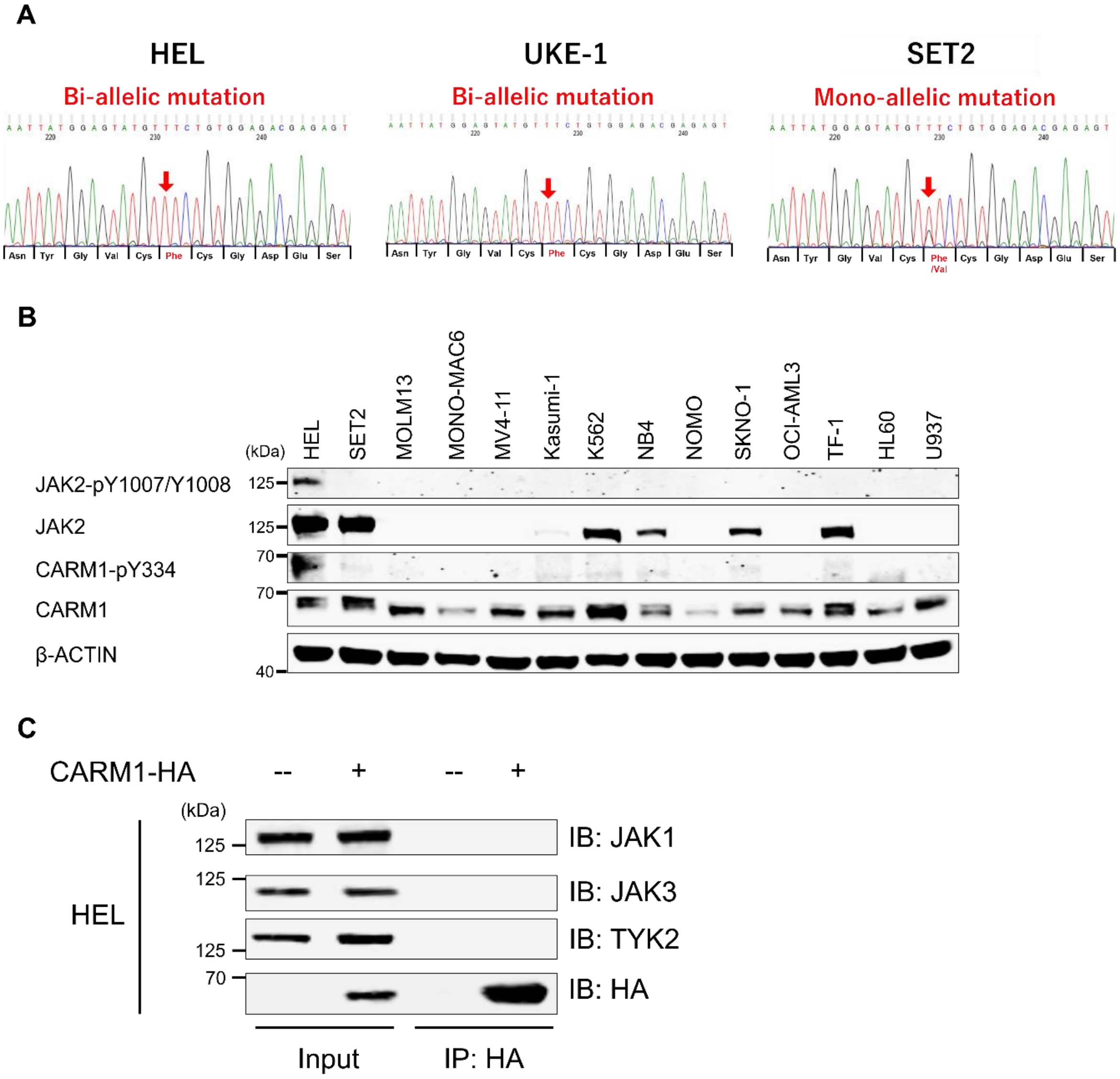

Supplementary Figure 3. Mutational status of *JAK2*-V617F and JAK2 auto-phosphorylation

(A) *JAK2*-V617F mutation was detected by direct sequencing after the amplification of genomic DNA by PCR as described previously [1]. Positions of the *JAK2*-V617F mutation are indicated by arrows. HEL and UKE-1 cells have bi-allelic mutations (homozygous), while SET2 cells has a mono-allelic mutation (heterozygous) at the genomic DNA level.

(B) The phosphorylation status of JAK2 and CARM1 is shown in 14 myeloid leukemia cell lines. HEL cells showed the highest level of JAK2 auto-phosphorylation.

(C) Immunoprecipitation was performed using HEL cells that express HA-tagged CARM1, with an anti-HA antibody. Immunoblotting with anti-HA and anti-JAK1, JAK2, and TYK2 antibodies did not identify any binding of CARM1 to JAK1, JAK2, and TYK2.

Supplementary Figure 4.

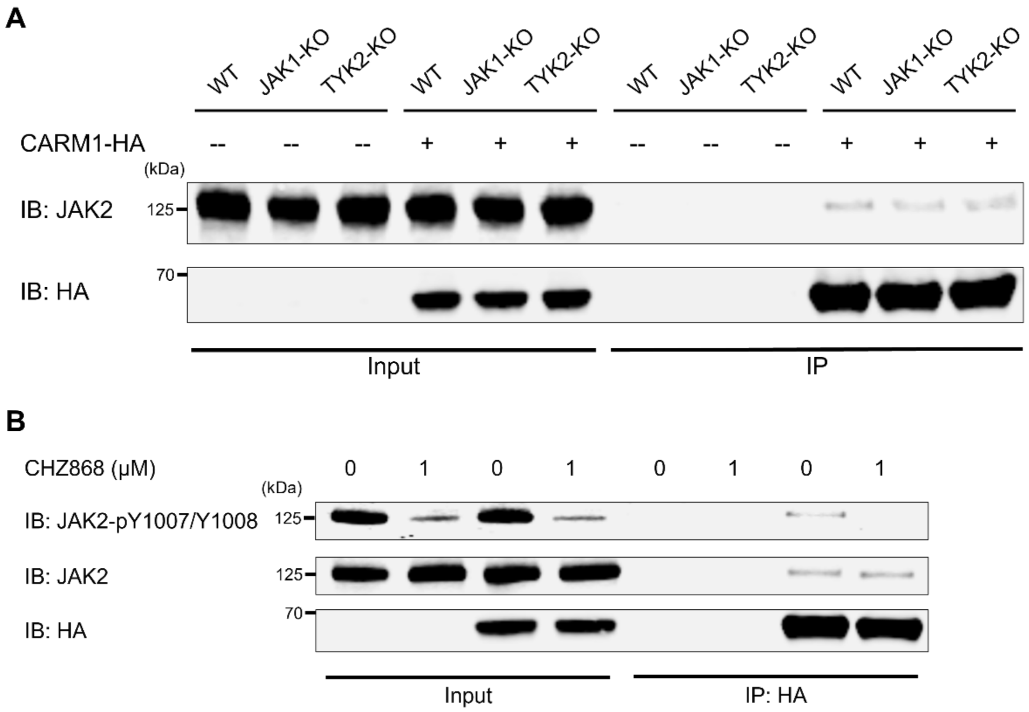

Supplementary Figure 4. The effect of transphosphorylation of JAK2 by JAK1 or TYK2 on the binding between JAK2 and CARM1

(A) Immunoprecipitation was performed using clustered regularly interspaced short palindromic repeat (CRISPR)/CRISPR-associated protein-9 (Cas9) mediated JAK1 or TYK2 knockout HEL cells that express HA-tagged CARM1, with an anti-HA antibody. Immunoblotting with anti-HA and anti-JAK2 antibodies revealed the interaction between JAK2 and CARM1. Knockout of JAK1 or TYK2 did not alter the binding between CARM1 and JAK2.

(B) Immunoprecipitation was performed with an anti-HA antibody using HEL cells expressing HA-tagged CARM1 which were treated with type II JAK2 inhibitor (CHZ868) for 24 hours. Immunoblotting with anti-HA and anti-JAK2 antibodies revealed the interaction between JAK2 and CARM1. The binding between CARM1 and JAK2 was observed after reduction of JAK2 auto-phosphorylation.

Supplementary Figure 5.

A

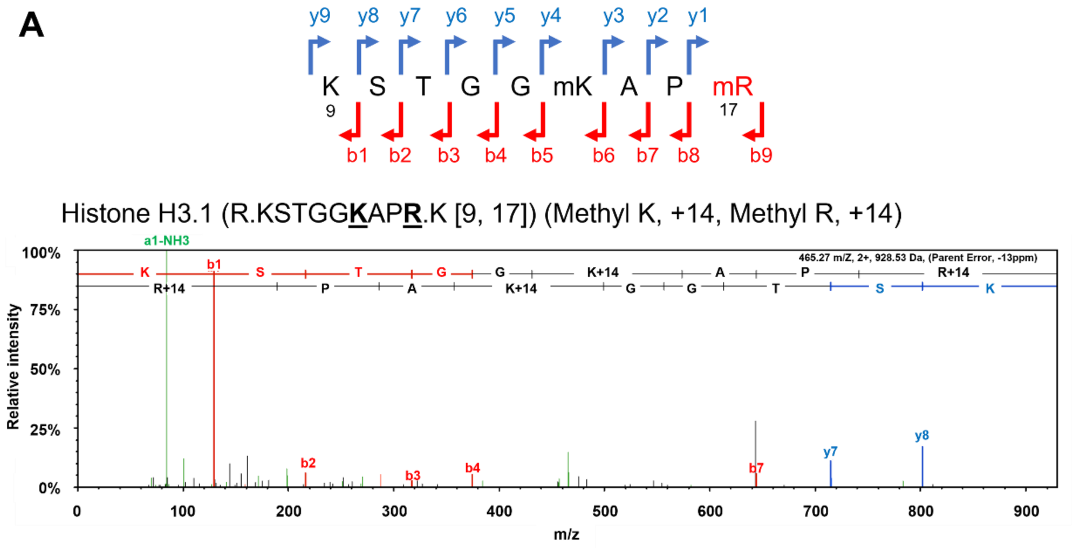

	Ion signal (m/z)	Amino acids	Ion signal (m/z)	
b1	129.1	K	926.6	y9
b2	216.1	S	801.5	y8
b3	317.2	T	714.4	y7
b4	374.2	G	613.4	y6
b5	431.2	G	556.4	y5
b6	573.3	K methylation	499.3	y4
b7	644.4	A	357.2	y3
b8	741.4	P	286.2	y2
b9	929.6	R methylation	189.1	y1

Fragments with 1 methylated residue (b6–b8)

Fragments with 2 methylated residues (b9)

Fragments with 2 methylated residues (y4–y9)

Fragments with 1 methylated residue (y1–y3)

B

	Relative methylation intensity of Histone H3.1 (KSTGGKAPR)
With non-phosphorylated CARM1	3.66×10^{6}
With phosphorylated CARM1	1.58×10^{7}

Supplementary Figure 5. Mass spectrometry for the substrates of CARM1 after *in vitro* methylation assay

(A) Recombinant CARM1 was incubated with or without active JAK2 kinase in an *in vitro* assay, then phosphorylated CARM1 or non-phosphorylated CARM1 were subsequently incubated with histone 3.1. These substrates were subjected to LC-MS/MS analysis. Histone 3.1 peptides containing methylated R17 were identified in the incubation with phosphorylated CARM1 and non-phosphorylated CARM1.

(B) The number of histone H3.1 molecules with methylated residues was increased following incubation with phosphorylated CARM1 compared to non-phosphorylated CARM1.

Supplementary Figure 6.

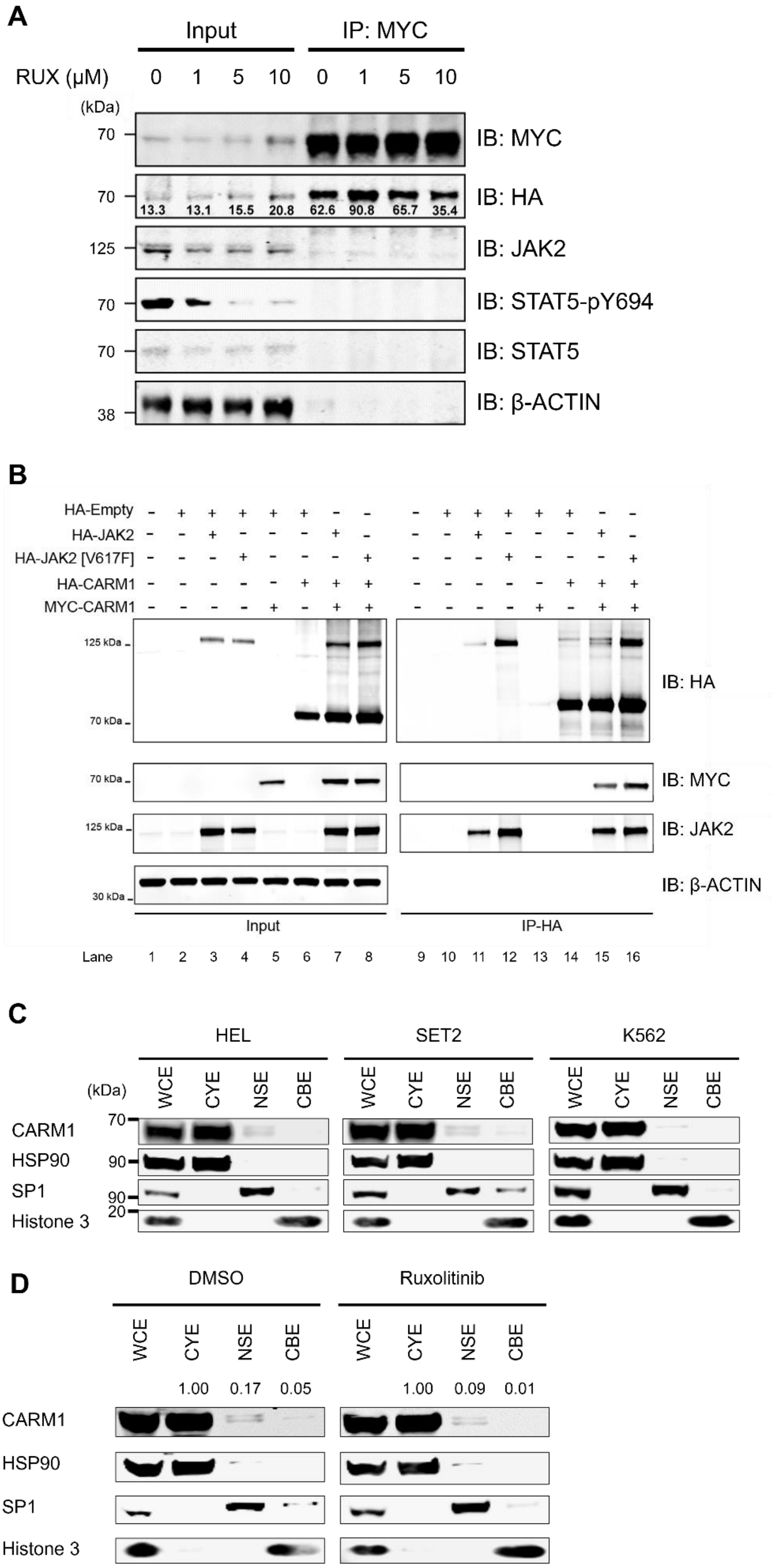

Supplementary Figure 6. Dimerization and subcellular localization of CARM1

(A) HA-tagged WT-CARM1 and MYC-tagged WT-CARM1 constructs were co-transfected into 293T cells. Following immunoprecipitation with anti-MYC antibody, the detectable HA-tagged WT-CARM1 was decreased in the 293T cells treated with RUX 10 μM *vs.* those treated with DMSO (RUX 0 μM).

(B) HA-tagged WT-CARM1 and MYC-tagged WT-CARM1 constructs were co-transfected into 293T cells overexpressing HA-tagged WT-JAK2 or JAK2-V617F mutants. Following immunoprecipitation with anti-HA antibody, the detectable MYC-tagged WT-CARM1 was increased in the 293T cells expressing JAK2-V617F (lane 16) *vs.* those expressing WT-JAK2 (lane 15).

(C) The immunoblotting assay of subcellular localizations in HEL, SET2, and K562 cells revealed that total CARM1 is localized mainly in the cytoplasm. Purity of the subcellular fractions was assessed by detecting HSP90 (cytoplasmic extraction, CYE), SP1 (nuclear soluble extraction, NSE), and histone H3 (chromatin-bound extract, CBE). WCE indicates proteins within the whole-cell lysates.

(D) Immunoblotting to assess CARM1 subcellular localization in HEL cells treated with DMSO or ruxolitinib (RUX) (500 nM).

Supplementary Figure 7.

A

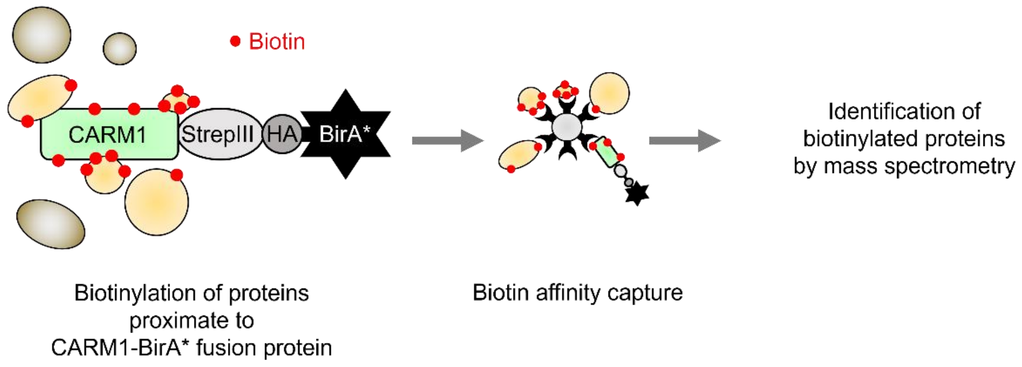

B

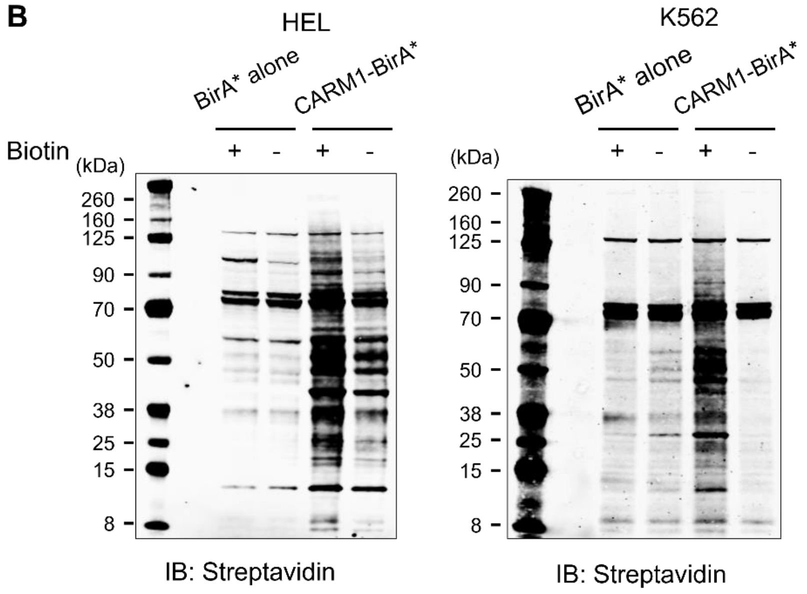

C

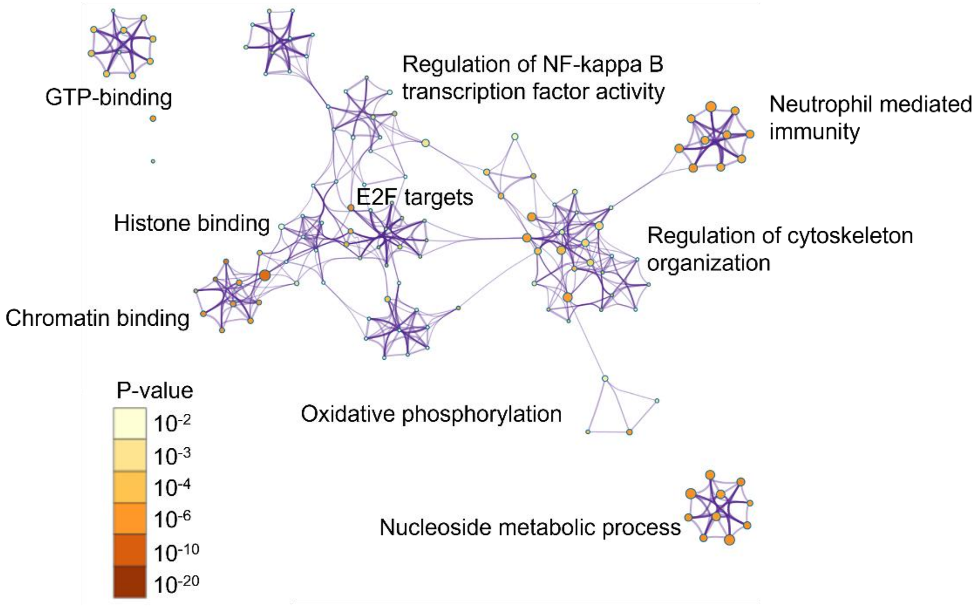

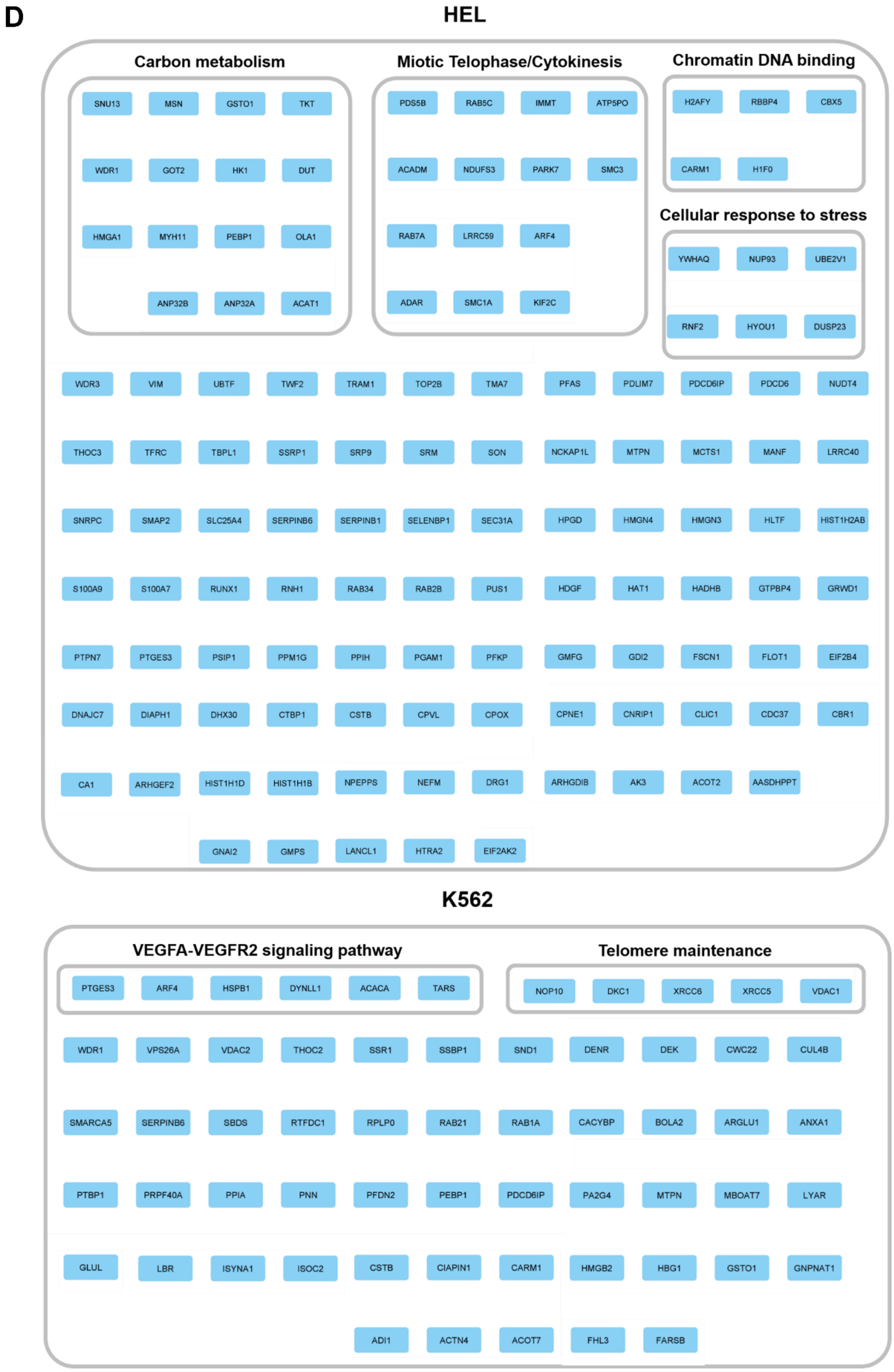
D
HEL
Carbon metabolism
SNU13 MSN GSTO1 TKT
WDR1 GOT2 HK1 DUT
HMGA1 MYH11 PEBP1 OLA1
ANP32B ANP32A ACAT1
Miotic Telophase/Cytokinesis
PDS5B RAB5C IMMT ATP5PO
ACADM NDUFS3 PARK7 SMC3
RAB7A LRRC59 ARF4
ADAR SMC1A KIF2C
Chromatin DNA binding
H2AFY RBBP4 CBX5
CARM1 H1F0
Cellular response to stress
YWHAQ NUP93 UBE2V1
RNF2 HYOU1 DUSP23
WDR3 VIM UBTF TWF2 TRAM1 TOP2B TMA7 PFAS PDLIM7 PDCD6IP PDCD6 NUDT4
THOC3 TFRC TBPL1 SSRP1 SRP9 SRM SON NCKAP1L MTPN MCTS1 MANF LRRC40
SNRPC SMAP2 SLC25A4 SERPINB6 SERPINB1 SELENBP1 SEC31A HPGD HMGN4 HMGN3 HLTF HIST1H2AB
S100A9 S100A7 RUNX1 RNH1 RAB34 RAB2B PUS1 HDGF HAT1 HADHB GTPBP4 GRWD1
PTPN7 PTGES3 PSIP1 PPM1G PPIH PGAM1 PFKP GMFG GDI2 FSCN1 FLOT1 EIF2B4
DNAJC7 DIAPH1 DHX30 CTBP1 CSTB CPVL CPOX CPNE1 CNRIP1 CLIC1 CDC37 CBR1
CA1 ARHGEF2 HIST1H1D HIST1H1B NPEPPS NEFM DRG1 ARHGDIB AK3 ACOT2 AASDHPPT
GNAI2 GMPS LANCL1 HTRA2 EIF2AK2
K562
VEGFA-VEGFR2 signaling pathway
PTGES3 ARF4 HSPB1 DYNLL1 ACACA TARS
Telomere maintenance
NOP10 DKC1 XRCC6 XRCC5 VDAC1
WDR1 VPS26A VDAC2 THOC2 SSR1 SSBP1 SND1 DENR DEK CWC22 CUL4B
SMARCA5 SERPINB6 SBDS RTFDC1 RPLP0 RAB21 RAB1A CACYBP BOLA2 ARGLU1 ANXA1
PTBP1 PRPF40A PPIA PNN PFDN2 PEBP1 PDCD6IP PA2G4 MTPN MBOAT7 LYAR
GLUL LBR ISYNA1 ISOC2 CSTB CIAPIN1 CARM1 HMGB2 HBG1 GSTO1 GNPNAT1
ADI1 ACTN4 ACOT7 FHL3 FARSB

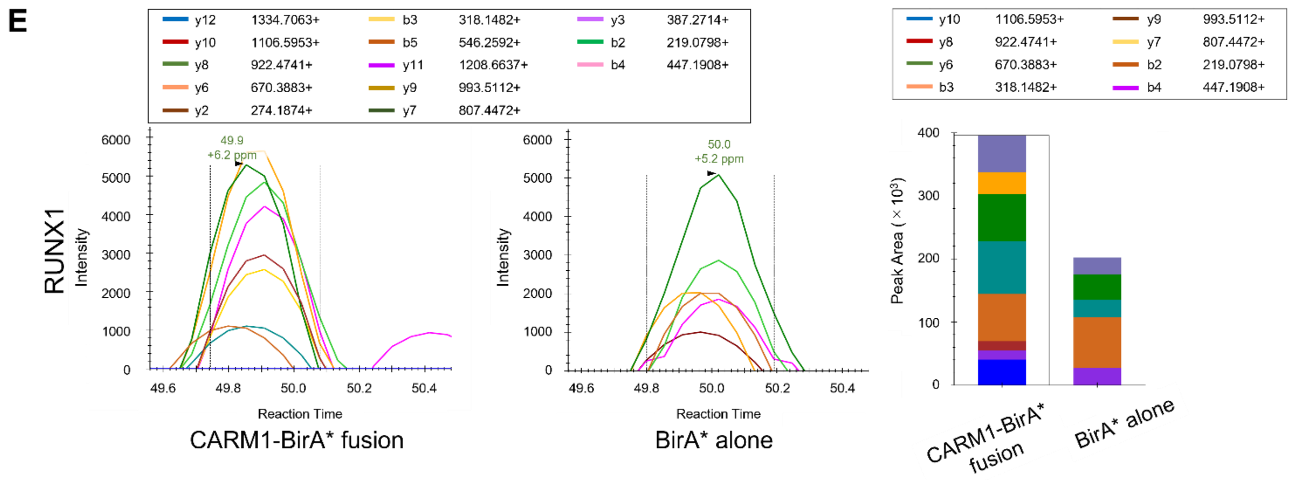

Supplementary Figure 7. The interactome of CARM1 by BioID system

(A) Schematic diagram demonstrating BioID (proximity-dependent biotin identification) approach for the identification of CARM1-interacting proteins.

(B) Western blots confirming biotinylated proteins of lysate from HEL cells with CARM1-BirA* fusion proteins overexpression in culture medium with or without biotin.

(C) Metascape enrichment network visualization showing the intra-cluster and inter-cluster similarities of enrichment terms, up to nine terms per clusters, in HEL cells. Terms are defined according to GO/KEGG terms, canonical pathways, and hallmark gene sets. The connecting pairs of nodes are created with Kappa score >0.3. Terms containing more genes tend to have a more significant *P*-value; the darker color of the node indicates the more statistically significant *P*-value. CARM1-associated proteins clustered into groups of proteins identified as being involved in purine nucleotide metabolism, E2F targets, MYC targets, histone and chromatin binding proteins, and ribonucleoprotein complex biogenesis, based on enriched pathway analysis of Metascape (metascape.org) for both cell lines.

(D) Proteins binding to CARM1 and enrichment network visualization for the results in HEL and K562 cells. See also supplementary data. As JAK2 are weakly and transiently associated with its substrates, we did not observe the interaction of CARM1 with JAK2 in HEL cells.

(E) Targeted mass spectrometry analysis using parallel reaction monitoring revealed the interaction of CARM1 with RUNX1 in HEL cells. Skyline chromatogram visualizations showed the intensity at each resampled retention time point for all fragment ions, which were presented as different colored lines identified in the legends. Peak areas by each fragment were displayed as the bar graphs (left) to compare the intensity of samples from HEL cells with CARM1-BirA* fusion (total AUC value, 395,628) to those with BirA* alone (total AUC value, 215,928).

Supplementary Figure 8.

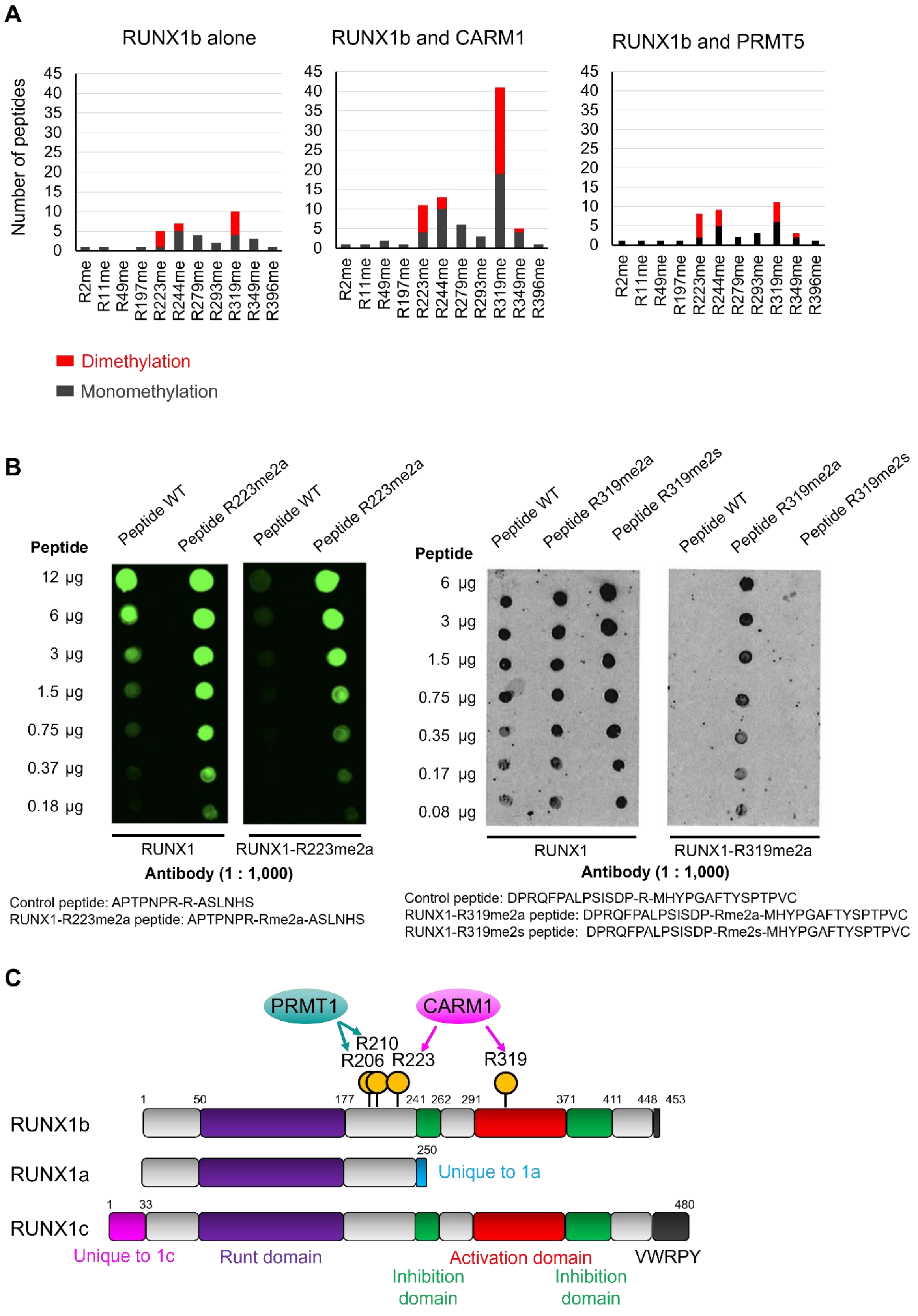

D

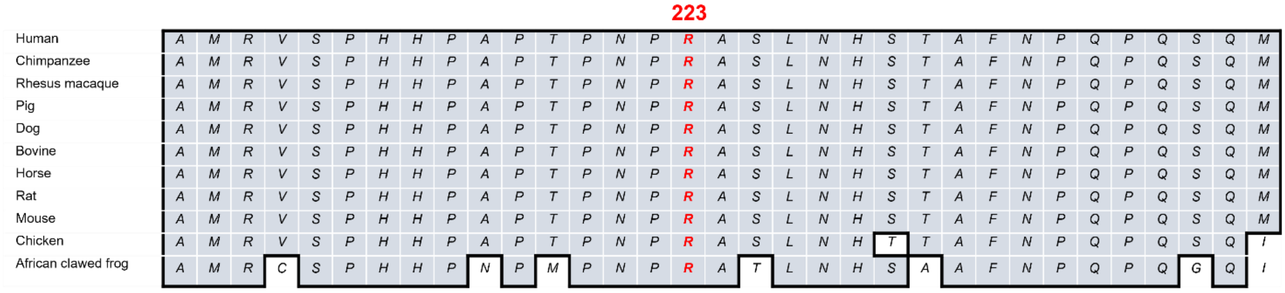

E

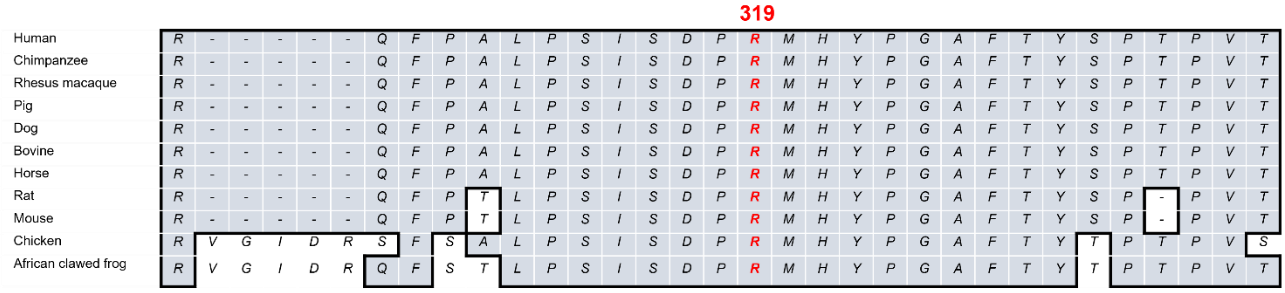

Supplementary Figure 8. Asymmetric dimethylation of R223 and R319 in RUNX1b

(A) Recombinant RUNX1b (full-length) was generated from baculovirus-infected Sf9 cells. Recombinant RUNX1b was incubated with CARM1 or PRMT5 in an *in vitro* methylation assay, and RUNX1b peptides were subjected to LC-MS/MS analysis. The RUNX1b peptides containing methylated arginine-223 (R223) and arginine-319 (R319) were twice as represented in the incubation of RUNX1b and CARM1 than the RUNX1b alone (baseline). The asymmetric dimethylation levels of R223 and R319 on RUNX1b were comparable between RUNX1b only and incubation of RUNX1b with PRMT5.

(B) The dot-plot analysis showed that anti-RUNX1-R223 (left) or -R319 (right) methylation specific antibodies recognized methylated peptides in a dose-dependent manner.

(C) Schematic diagram of RUNX1b, showing the positions of R223 and R319; the numbers correspond to the amino acid positions.

(D) Sequence alignment around R223 of RUNX1b.

(E) Sequence alignment around R319 of RUNX1b. The alignments of R223 and R319 in RUNX1b showed are evolutionally conserved in mammals.

Supplementary Figure 9.

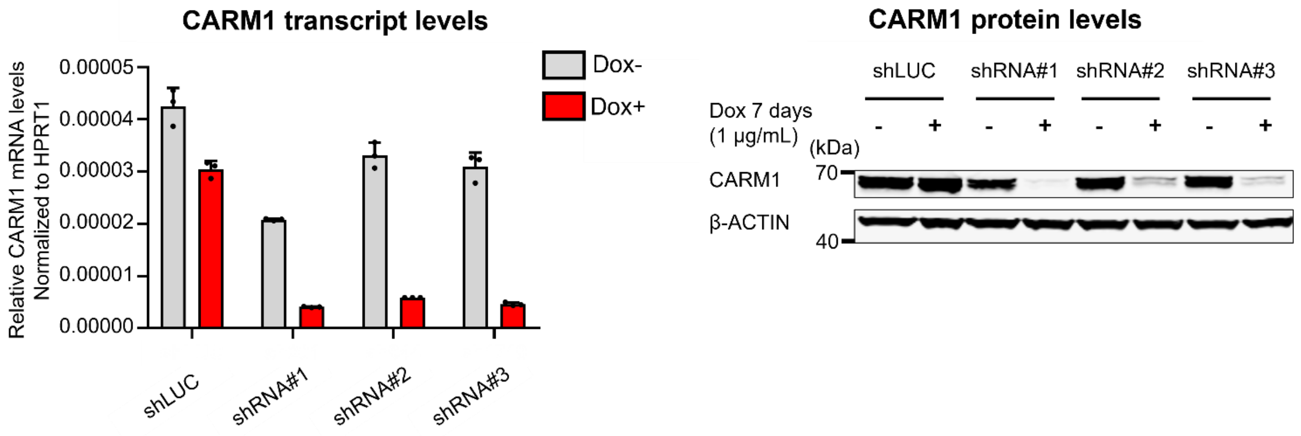

Supplementary Figure 9. Doxycycline-inducible shRNAs knockdown CARM1 in HEL cells
Doxycycline-inducible short hairpin RNAs (shRNAs) against CARM1 were stably expressed in HEL cells. CARM1 transcripts (left, n=3) and protein (right) were significantly reduced following 7 days of doxycycline exposure. All error bars represent the mean ± SD. All error bars represent the mean ± SD.

Supplementary Figure 10.

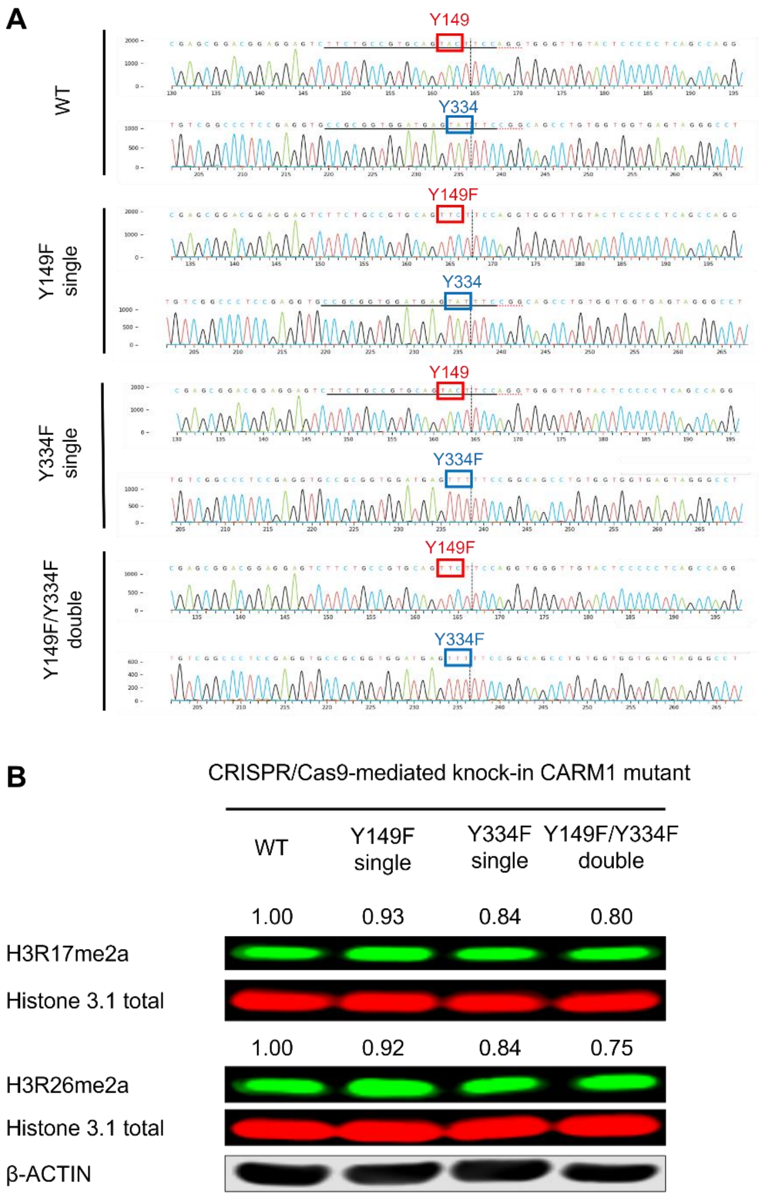

Supplementary Figure 10. Confirmation of non-phosphorylatable CARM1 mutation knock-in HEL cells

(A) Monoclonal populations of CRISPR/Cas9 mediated CARM1-Y149F or -Y334F single, or -Y149F/Y334F double mutant knock-in cells were generated after single cell expansion. Homozygous single mutations (Y149F or Y334F) or double mutations (Y149F/Y334F) were confirmed by direct sequencing after the amplification of genomic DNA.

(B) Clustered regularly interspaced short palindromic repeat (CRISPR)/CRISPR-associated protein-9 (Cas9)-mediated non-phosphorylatable CARM1 mutant knock-in modestly decreased the levels of ADMA histone H3R17 and H3R26.

Supplementary Figure 11.

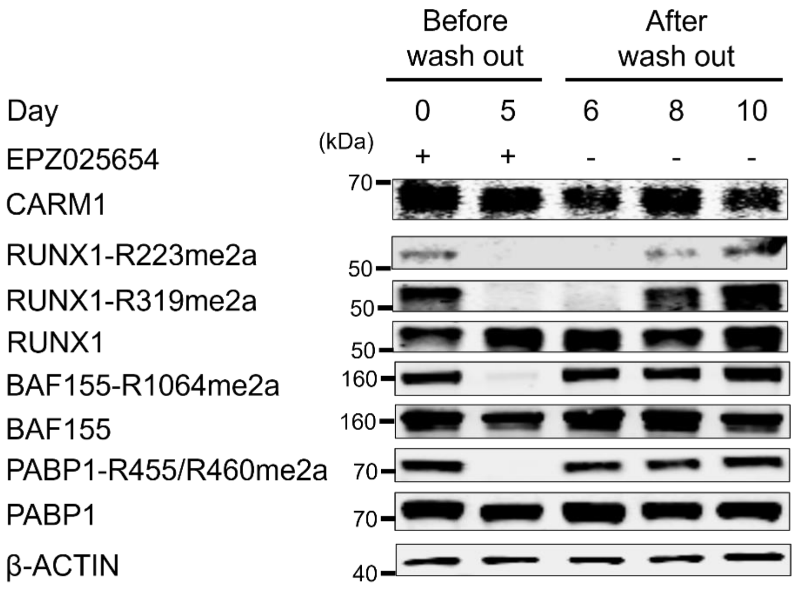

Supplementary Figure 11. The reversibility of ADMA in multiple CARM1 substrates

The asymmetrically dimethylated arginine levels of RUNX1, BAF155, and PABP1, were defined following EPZ025654 treatment for 5 days and a variable period of wash-out.

Supplementary Figure 12.

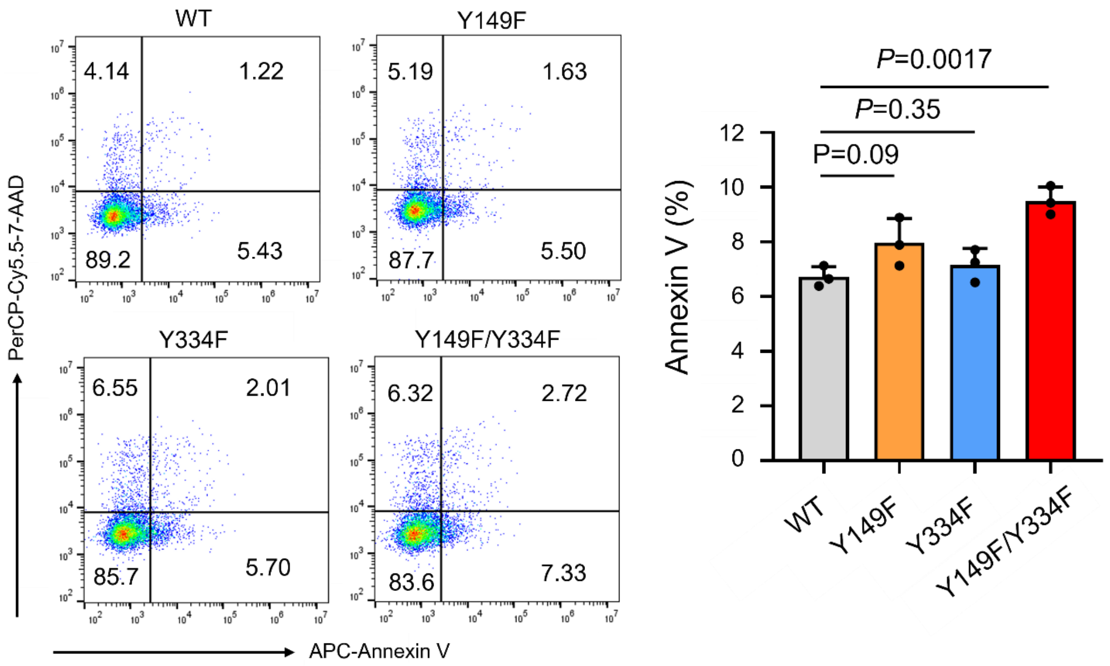

Supplementary Figure 12. Comparison of the effects of non-phosphorylatable CARM1 mutation on Annexin V/7-ADD positivity in HEL cells

Flow cytometric analysis of various CARM1 mutant HEL cells to detect annexin V positive cells (left panel). Fractions of the annexin V-positive populations are plotted (right panel) as mean ± s.d. n=3. All error bars represent the mean ± SD. *P* values were determined by two-tailed Student's t-test.

Supplementary Figure 13.

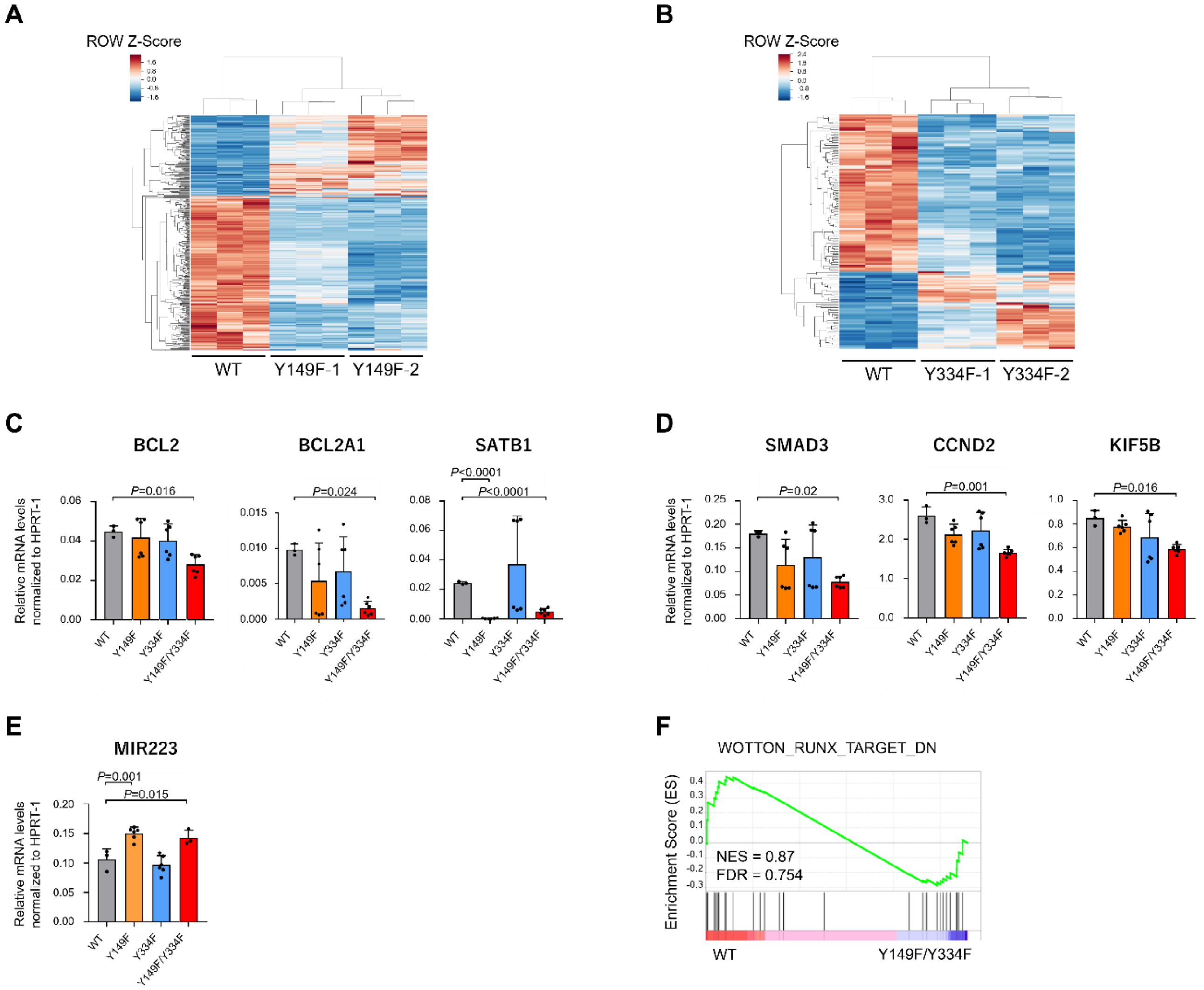

Supplementary Figure 13. Gene expression in non-phosphorylatable CARM1 mutation knock-in HEL cells

(A) Heatmap of the expression of 401 differentially expressed genes, representing replicates of cells expressing CARM1-WT or two independent cells expressing CARM1-Y149F single mutation (Y149F-1 and Y149F-2).

(B) Heatmap of the expression of 144 differentially expressed genes, representing replicates of cells expressing CARM1-WT or two independent cells expressing CARM1-Y334F single mutation (Y334F-1 and Y334F-2).

(C) qRT-PCR analysis showing expression genes associated with anti-apoptosis (*BCL2*, *BCL2A1*, and *SATB1*). Mean and SD are expressed as a percentage of *HPRT-1* expression, at least two biological replicates. CARM1 WT (n=3), Y149F (n=6), Y334F (n=6), and Y149F/Y334F mutation (n=6).

(D) qRT-PCR analysis showing expression genes associated with G2/M checkpoint (*SMAD3*, *CCND2*, and *KIF5B*). Mean and SD are expressed as a percentage of *HPRT-1* expression at least two biological

replicates. CARM1 WT (n=3), Y149F (n=6), Y334F (n=6), and Y149F/Y334F mutation (n=6).
(E) qRT-PCR analysis showing the higher expression of MIR223 in CARM1-Y149F/Y334F double mutation than CARM1-WT. Mean and SD are expressed as a percentage of *HPRT-1* expression at least two biological replicates. CARM1 WT (n=3), Y149F (n=6), Y334F (n=6), and Y149F/Y334F mutation (n=3).
(C)(D)(E) All error bars represent the mean ± SD. *P* values were determined by t one-way ANOVA followed by Dunnett's post hoc test.
(F) Representative GSEA plot depicting the regulation of "RUNX1-target" signature.

Supplementary Figure 14.

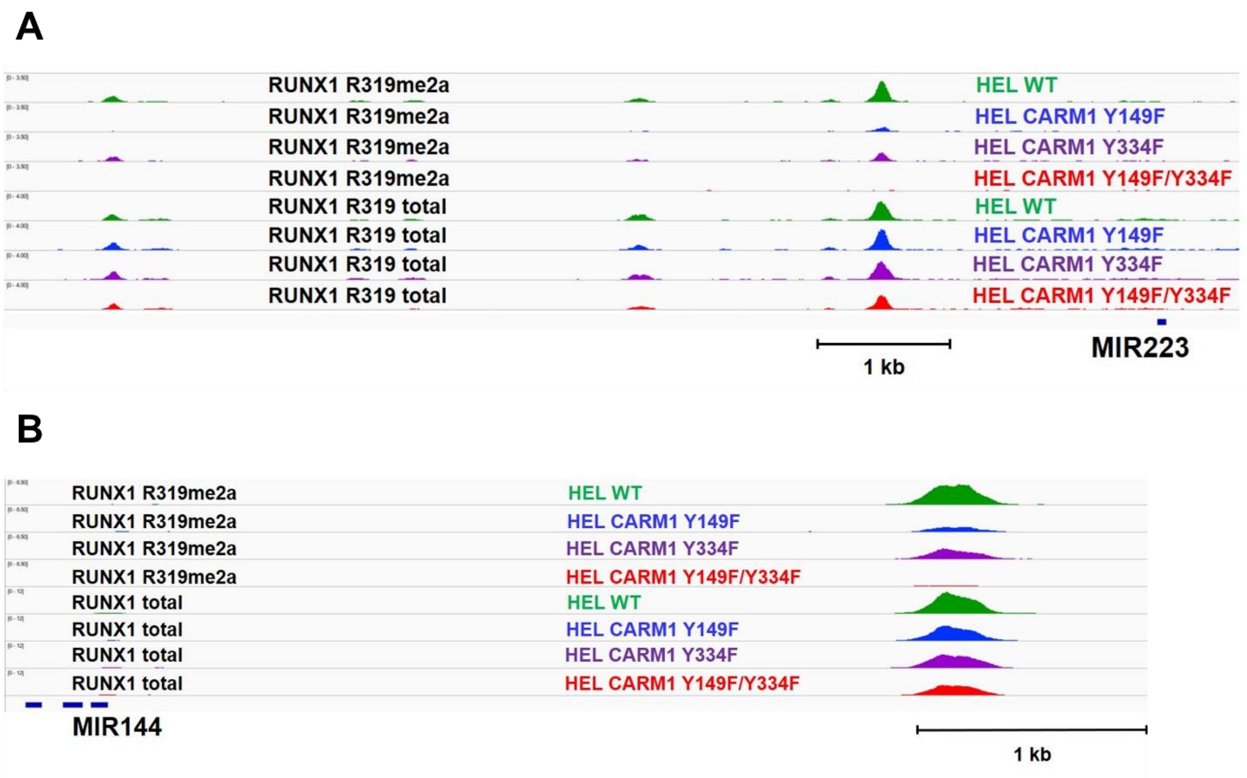

Supplementary Figure 14. ChIP-seq analysis demonstrating changes in asymmetrically dimethylated R319-RUNX1 occupancies on RUNX1 targeted genes

Target occupancies of RUNX1 and asymmetrically dimethylated R319-RUNX1 at *MIR144* (A) and *MIR223* genes (B) determined using antibody against total R319-RUNX1 and asymmetrically dimethylated R319-RUNX1, as shown in IGV genome browser tracks.

Supplementary Figure 15.

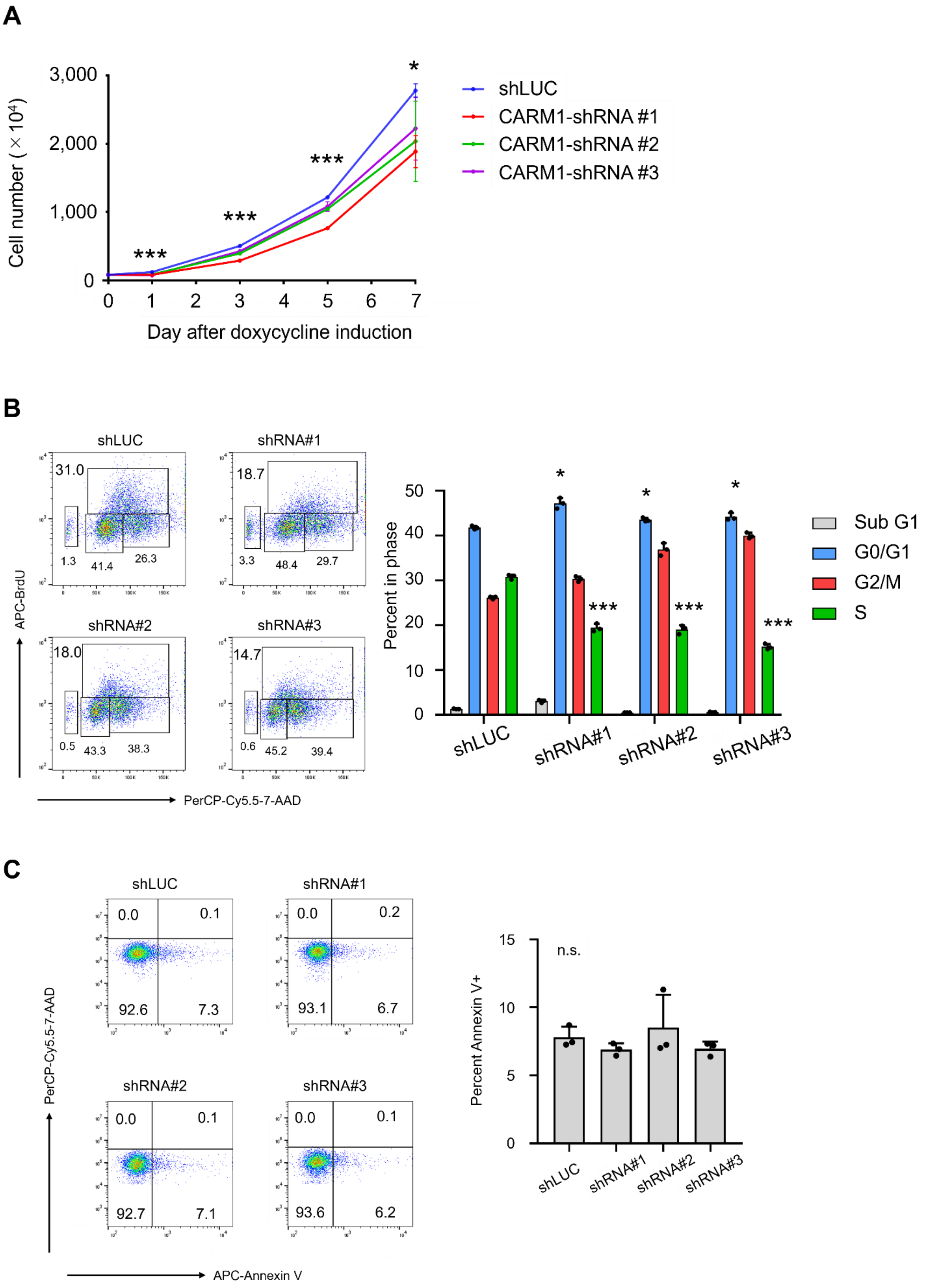

Supplementary Figure 15. The biological impact of CARM1 knockdown in HEL cells

(A) Cell counts from shRNA-mediated CARM1 knockdown (KD) HEL cells using three different shRNAs plus a control shRNA (shLUC). All error bars represent the mean ± SD. *P* values were determined by one-way ANOVA test.n=5, *p<0.05, ***P<0.001.

(B) Percentage of cells in sub-G1, G1, S or G2/M phase in HEL cells infected with a CARM1 KD lentivirus. All experiments were repeated three independent times. All error bars represent the mean ± SD. *P* values were determined by two-tailed Student's t-test. G0/G1 (p=0.002) and S populations (p<0.0001) in shRNA#1 vs. shLUC; G0/G1 (p=0.007) and S populations (p<0.0001) in shRNA#3 vs. shLUC; G0/G1 (p=0.012) and S populations (p<0.0001) in shRNA#3 vs. shLUC. *p<0.05, ***p<0.001.

(C) KD of CARM1 has minimal effect on apoptosis in HEL cells. The percentage of apoptotic cells in HEL cells stably expressing doxycycline-inducible shRNA against CARM1 are shown in bar graphs. All experiments were repeated three independent times. All error bars represent the mean ± SD. *P* values were determined by two-tailed Student's t-test

Supplementary Figure 16.

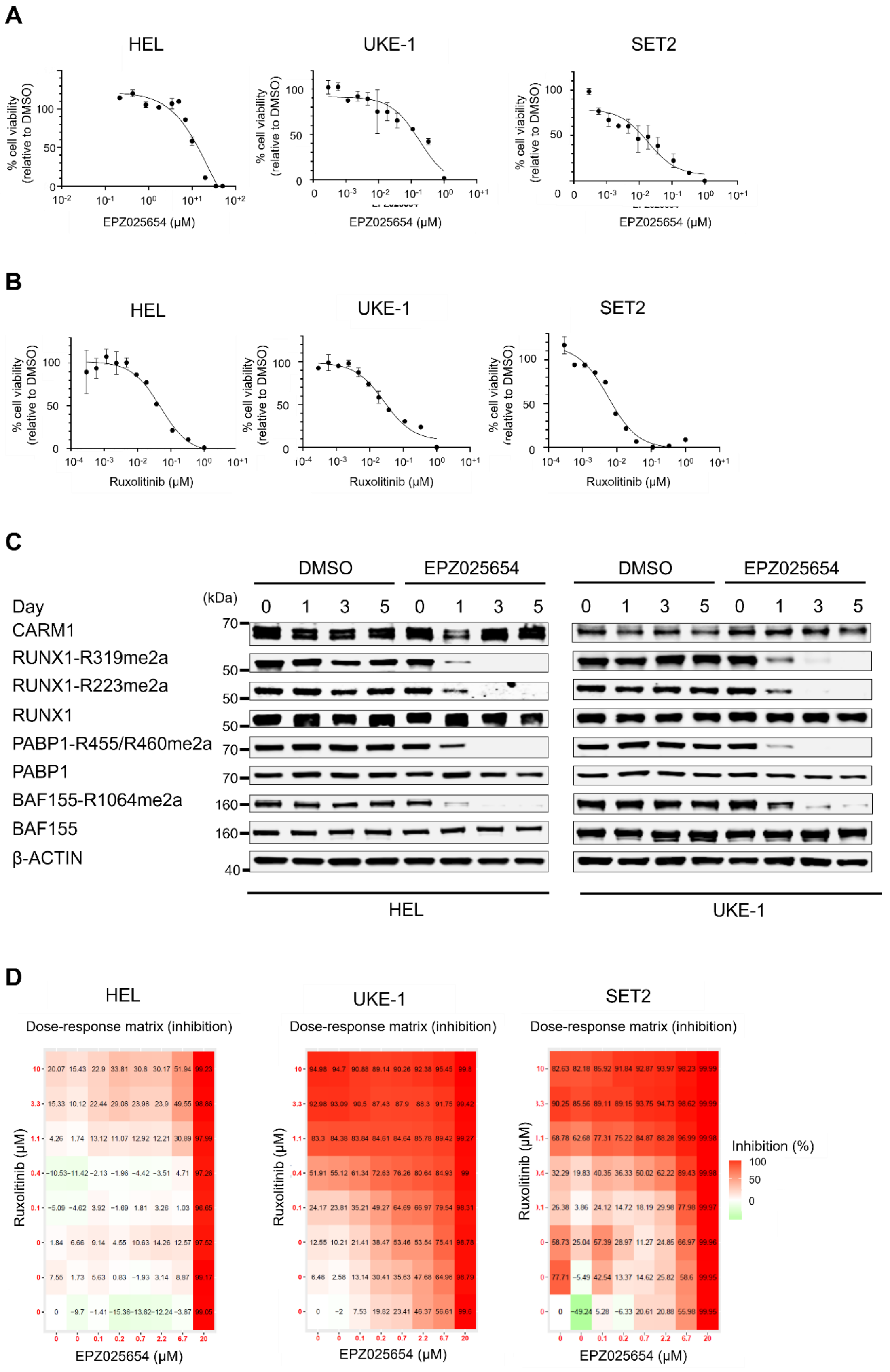

Supplementary Figure 16. Pharmacological inhibition of CARM1 and JAK2

(A) Proliferation of HEL, UKE-1, and SET2 cells (harboring JAK2-V617F mutations) with increasing concentration of EPZ025654 (μM) relative to DMSO. The half maximal inhibitory concentration (IC50) values for EPZ025654 are 23.7 μM, 1.8 μM, and 186.5 nM in HEL, UKE-1, and SET2 cells, respectively.

(B) Proliferation with increasing concentration of RUX (μM) relative to DMSO is depicted for HEL, UKE-1, and SET2 cells. IC50 values for RUX are 492 nM, 269 nM, and 56 nM in HEL, UKE-1, and SET2 cells, respectively.

(C) HEL cells were treated with a CARM1 inhibitor (5 μM EPZ025654) for 1, 3, and 5 days. The level of ADMA RUNX1, BAF155, and PABP1 was significantly decreased at day 1, and completely abolished at day 5.

(D) Visualization of the dose-response matrix and the plots of phenotypic cellular responses to EPZ025654, ruxolitinib, or the combination in HEL (left), UKE-1 (middle), and SET2 cells (right).

Supplementary Figure 17. Uncropped scans from figure 1 and 2. The boxes indicate the cropped images.

Figure 1A

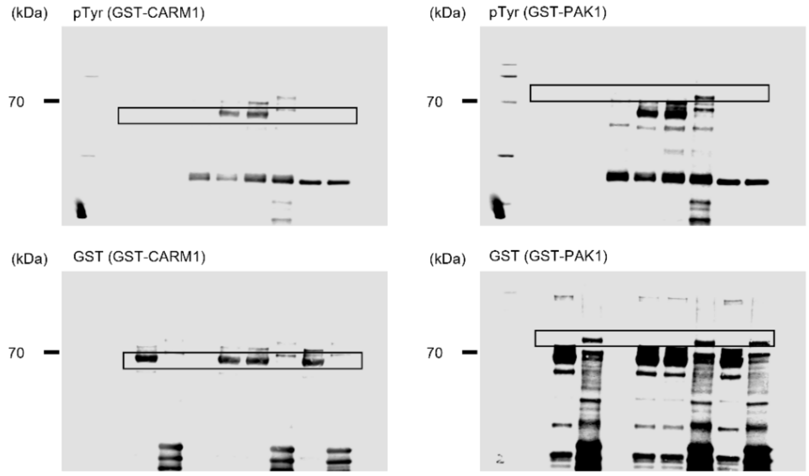

Figure 2A

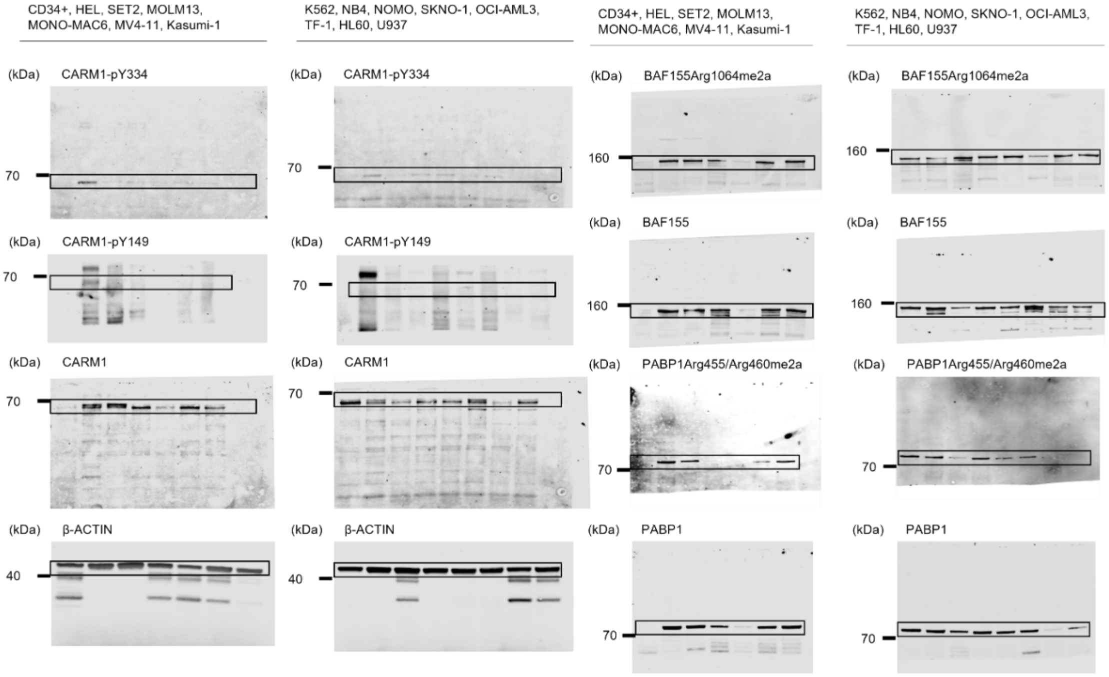

Figure 2B

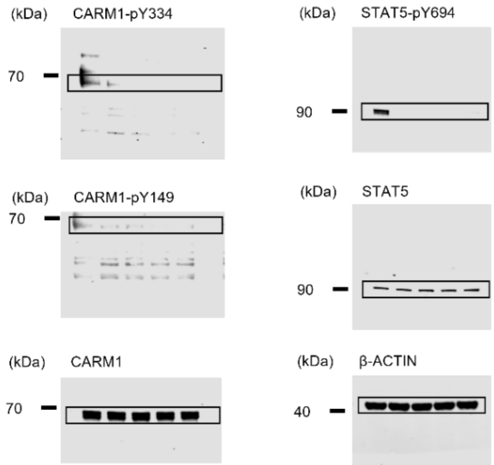

Figure 2C

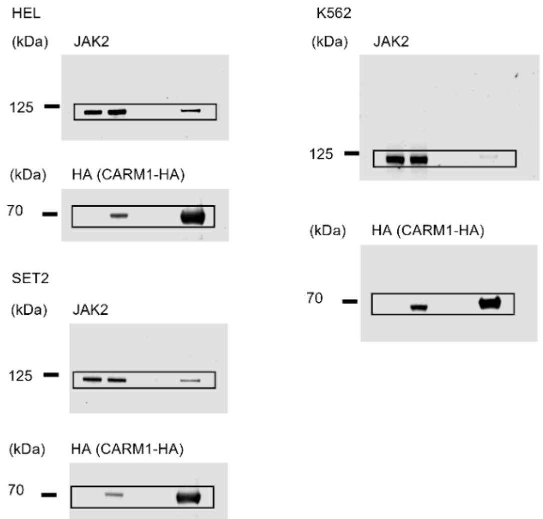

Figure 2D

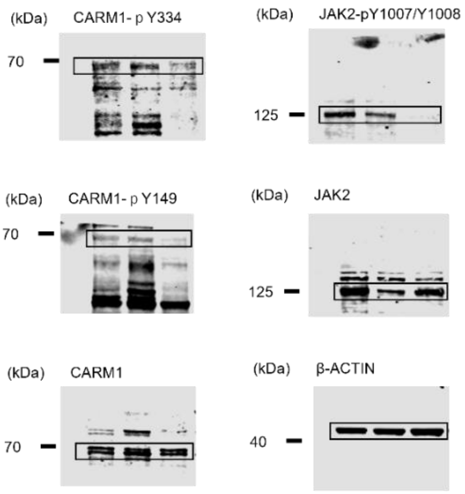

Figure 2E

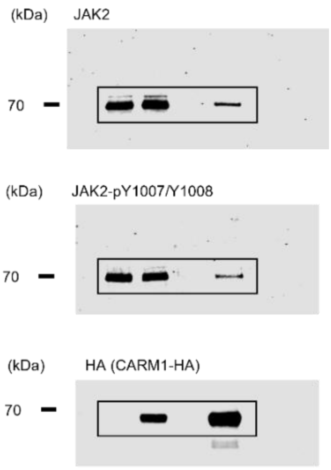

Figure 2F

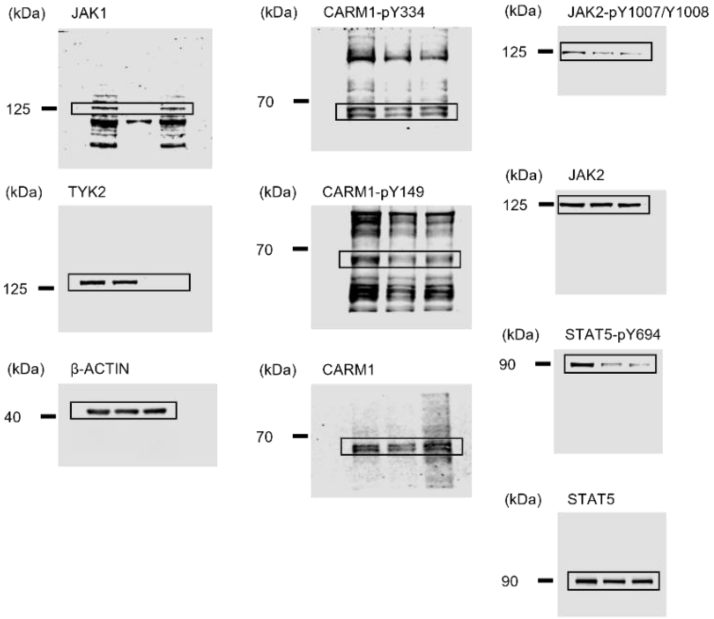

Figure 2G

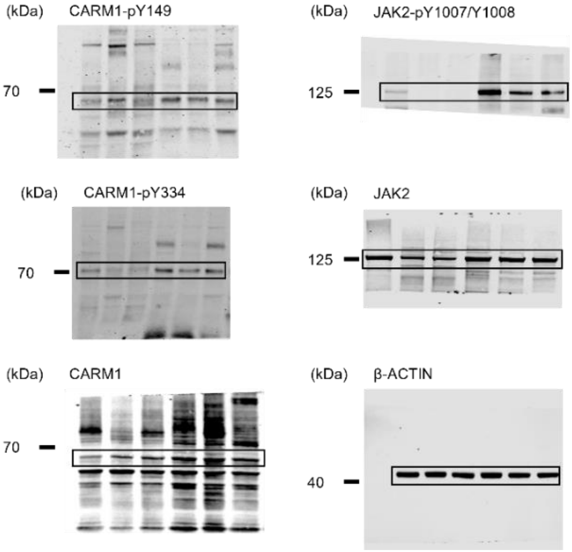

Supplementary Figure 18. Uncropped scans from figure 3. The boxes indicate the cropped images.

Figure 3C

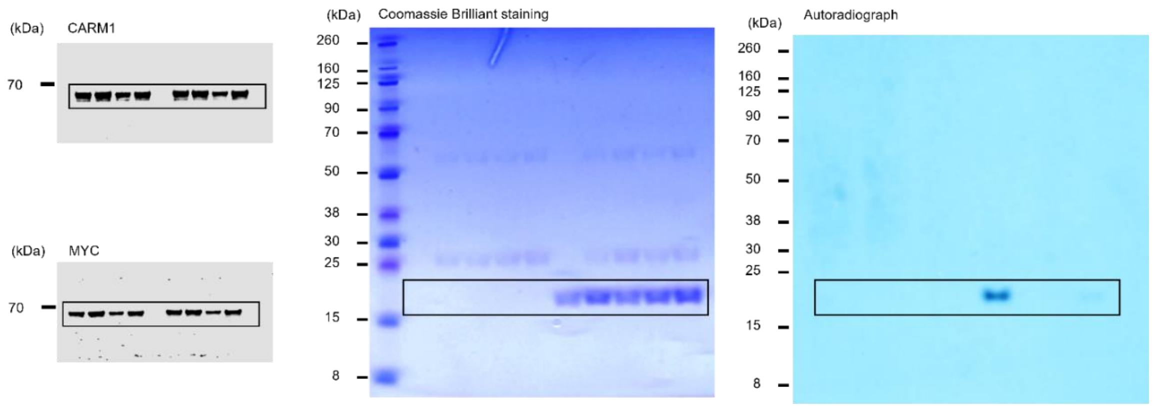

Figure 3D

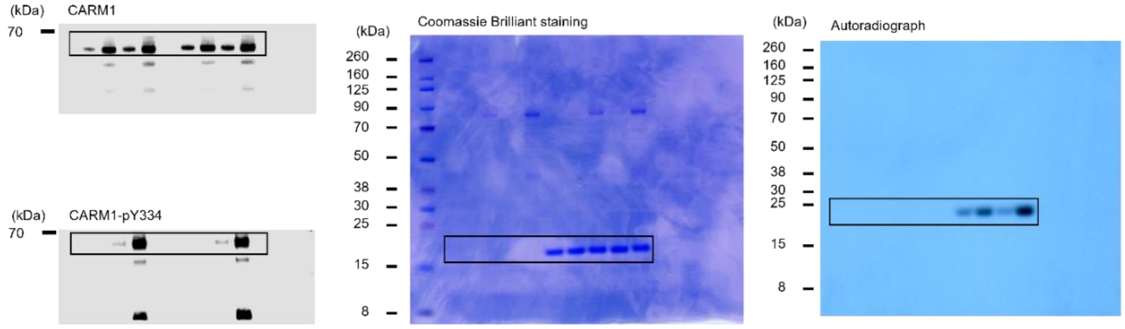

Figure 3F

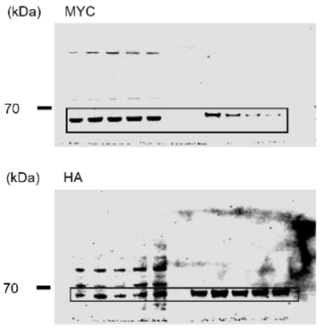

Figure 3G

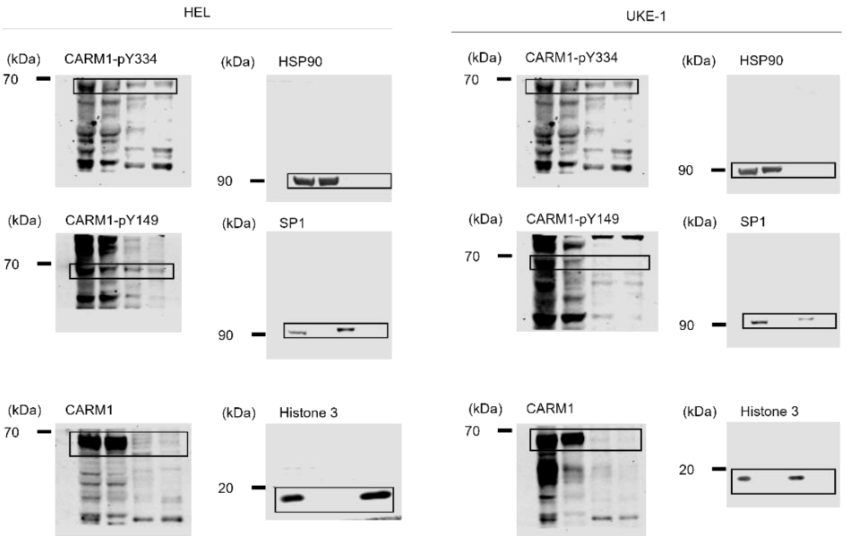

Supplementary Figure 19. Uncropped scans from figure 4. The boxes indicate the cropped images.

Figure 4B

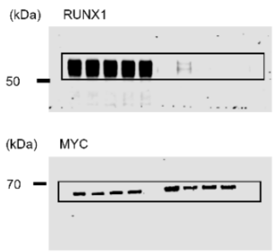

Figure 4C

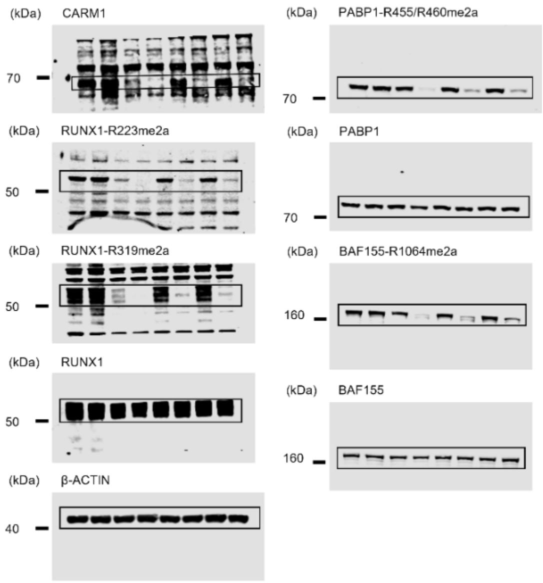

Figure 4D

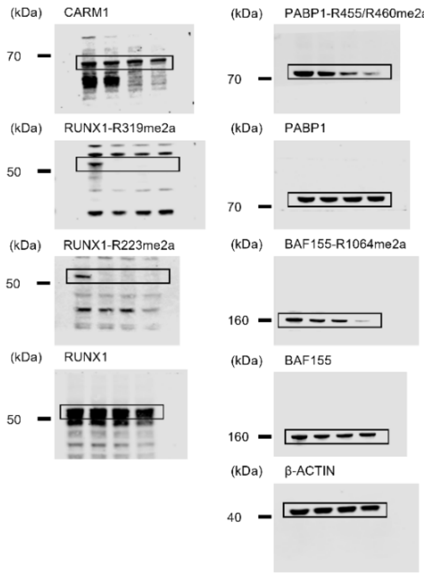

Figure 4E

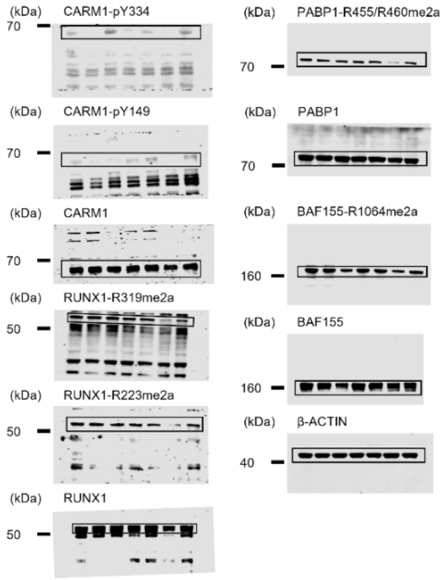

Figure 4F

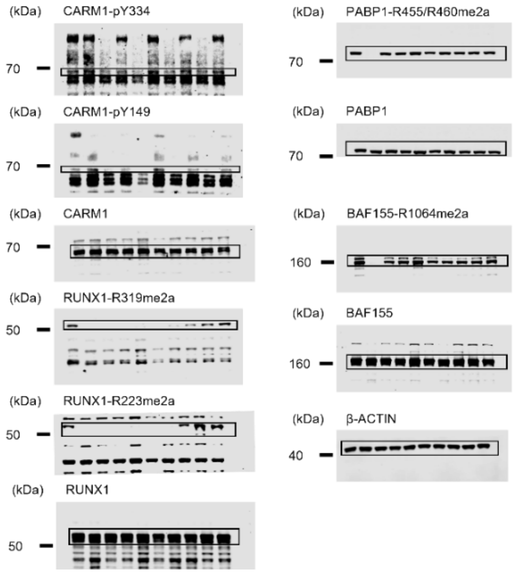

Supplementary Figure 20. Uncropped scans from figure 6. The boxes indicate the cropped images.

Figure 6A

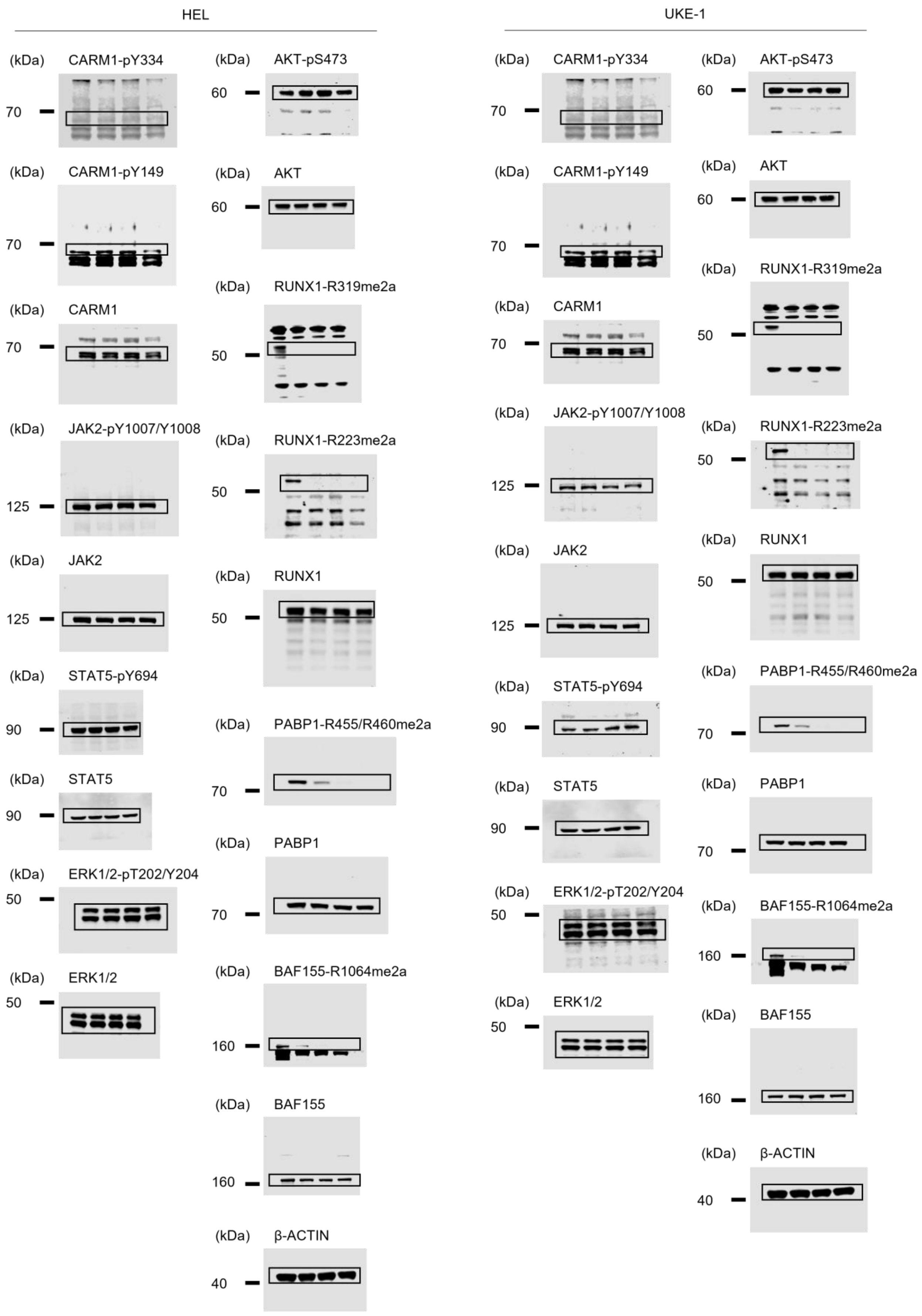

Supplementary Figure 21. Uncropped scans from supplementary figure 2, 3, and 4. The boxes indicate the cropped images.

Figure S2B

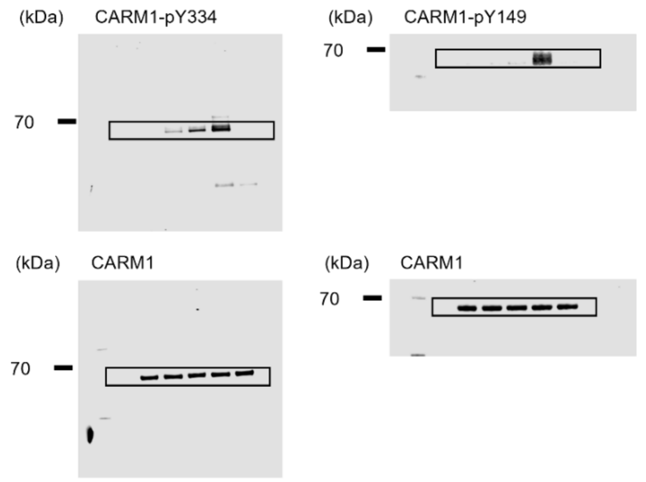

Figure S2D

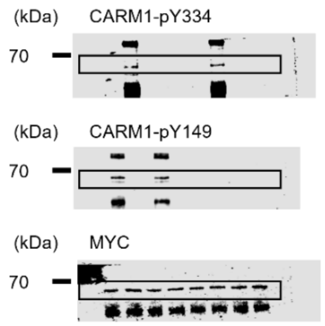

Figure S2F

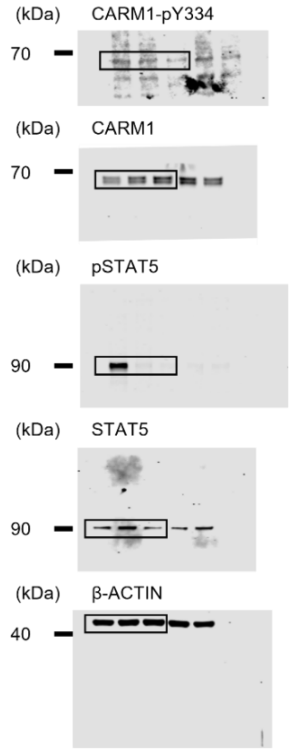

Figure S3B

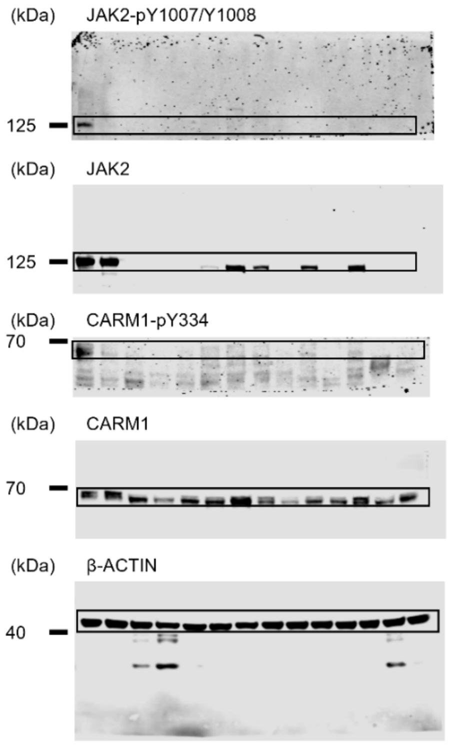

Figure S3C

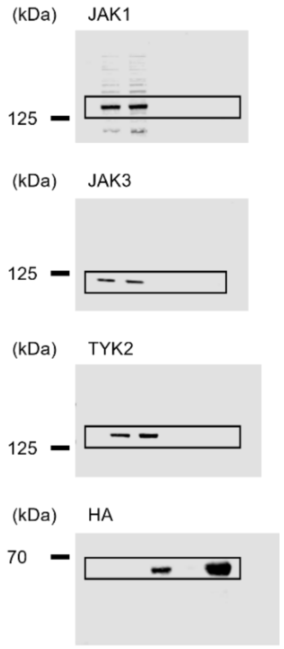

Figure S4A

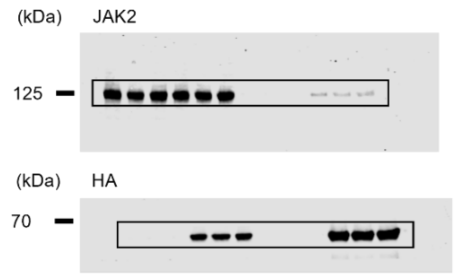

Figure S4B

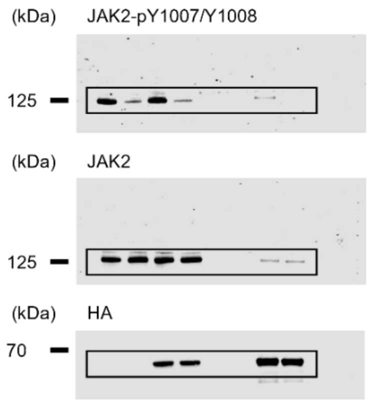

Supplementary Figure 22. Uncropped scans from supplementary figure 6. The boxes indicate the cropped images.

Figure S6A

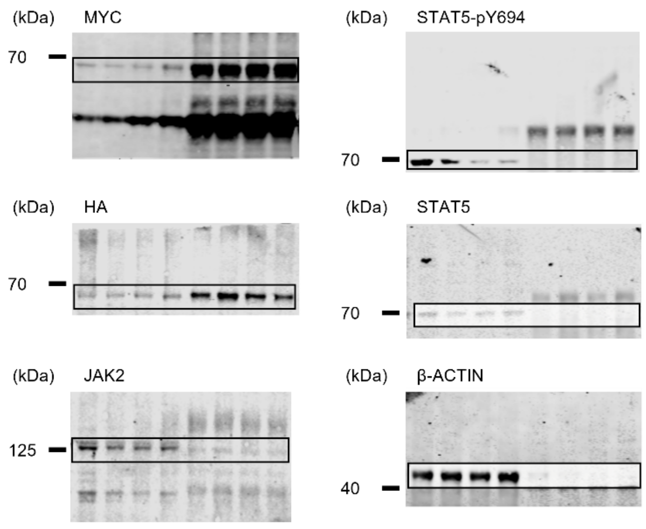

Figure S6B

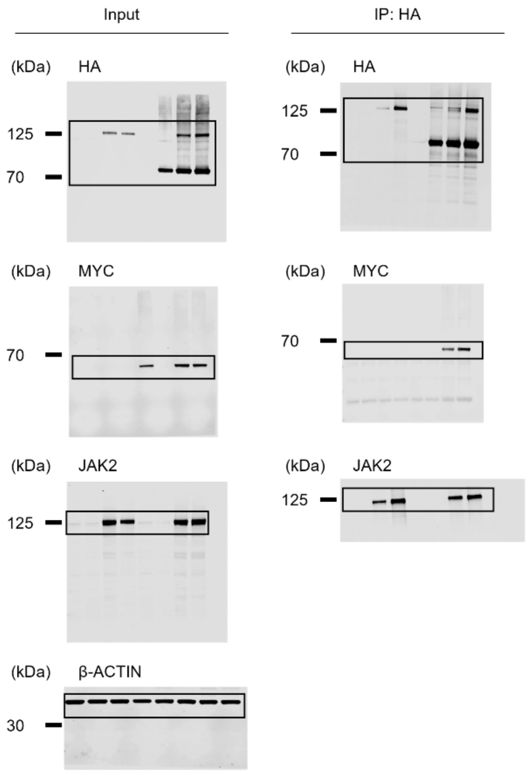

Figure S6C

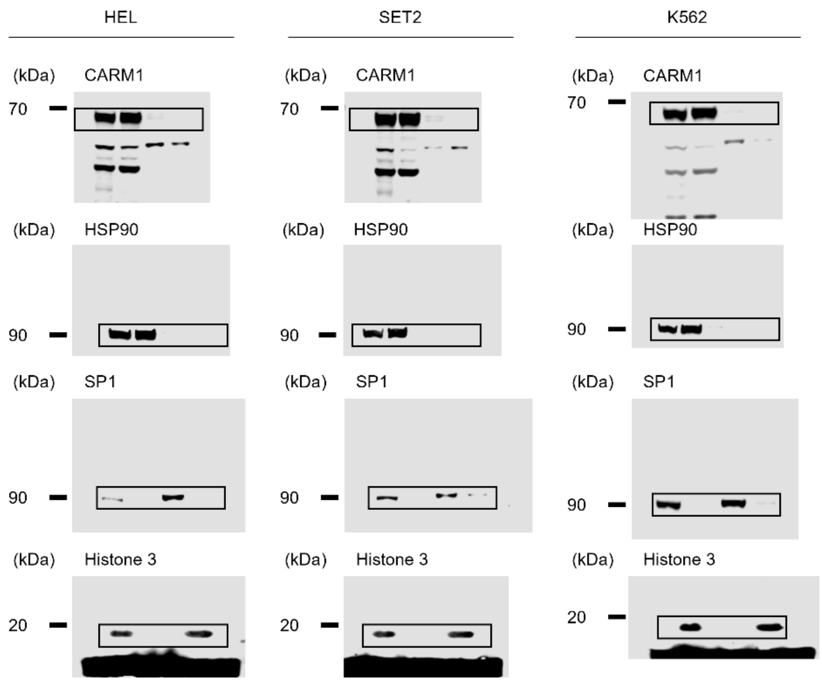

Figure S6D

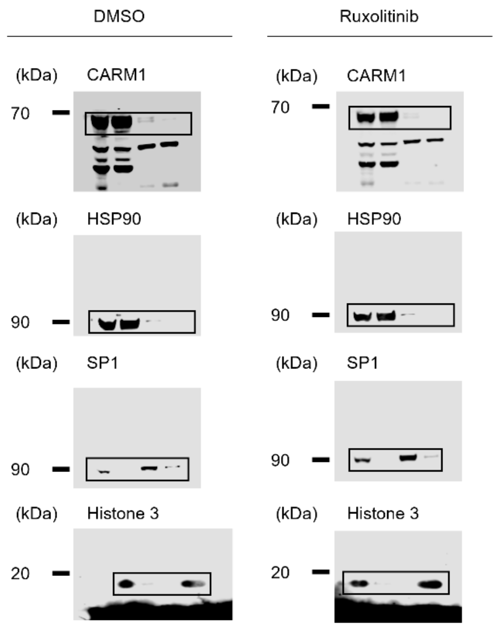

Supplementary Figure 23. Uncropped scans from supplementary figure 9, 10, 11, and 16. The boxes indicate the cropped images.

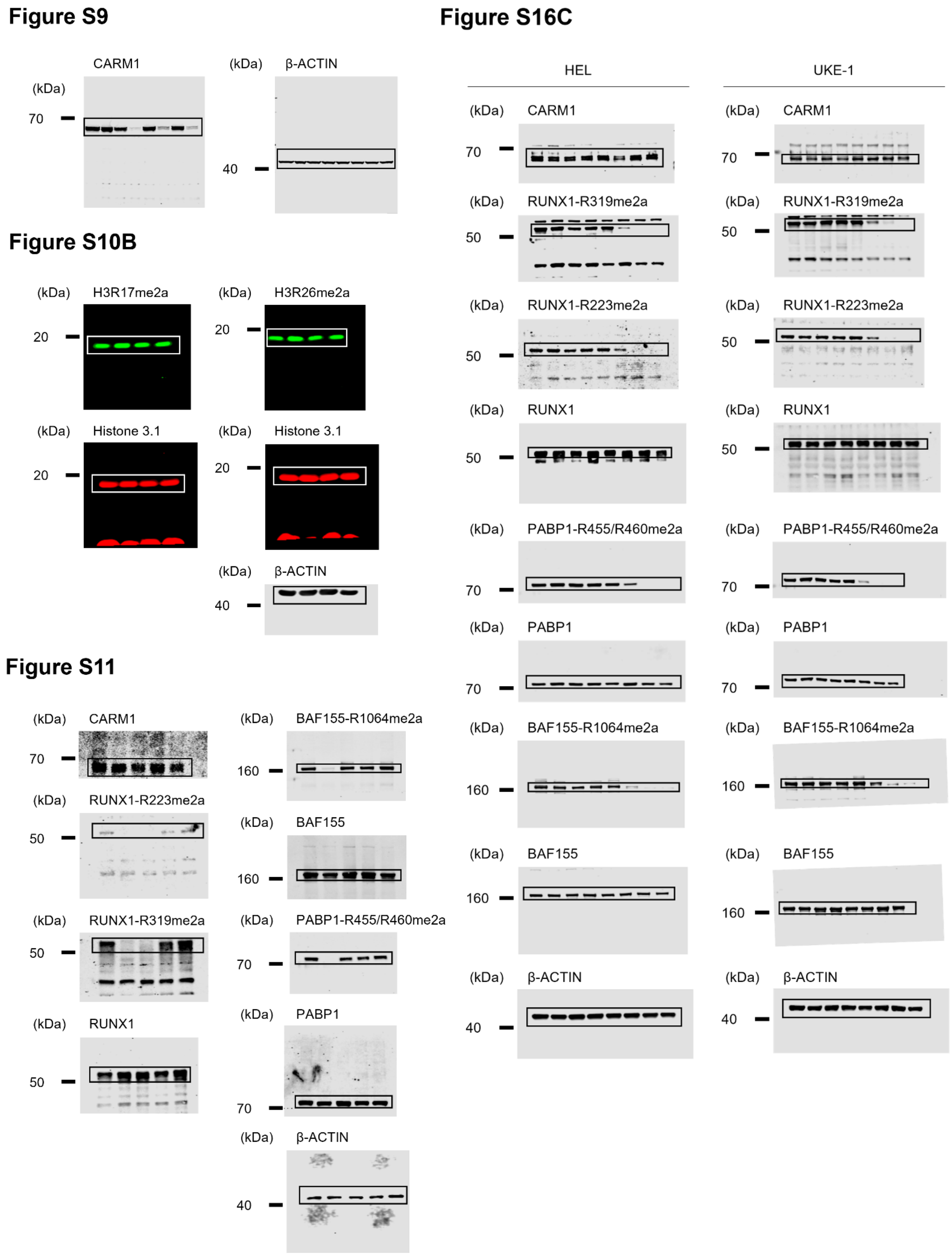

2. Supplementary Tables (Key resource table)

Supplementary Table 1. Leukemia cell lines

Cell line	Source	Identifier
Human: HEL	ATCC	TIB-180, RRID:CVCL_2481
Human: SET2	DSMZ	ACC-608, RRID:CVCL_2187
Human: MOLM-13	DSMZ	ACC-554, RRID:CVCL_RS50
Human: MONO-MAC6	DSMZ	ACC-124, RRID:CVCL 1426
Human: MV4-11	ATCC	CRL-9591, RRID:CVCL_0064
Human: Kasumi-1	ATCC	CRL-2724, RRID:CVCL_0589
Human: K562	ATCC	CCL-243, RRID:CVCL 0004
Human: NB-4	DSMZ	ACC-207, RRID:CVCL_0005
Human: NOMO-1	DSMZ	ACC-542, RRID:CVCL_1609
Human: SKNO-1	DSMZ	ACC-690, RRID:CVCL_2196
Human: OCI-AML3	DSMZ	ACC-582, RRID:CVCL_1844
Human: TF-1	ATCC	CRL-2003, RRID:CVCL_0559
Human: HL-60	ATCC	CCL-240, RRID:CVCL_A794
Human: U937	ATCC	CRL-1593.2, RRID:CVCL_0007
Human: UKE-1	Coriell	RRID:CVCL 0104
Human: CMK	DSMZ	ACC-392, RRID:CVCL 0216
Human: M-07e	DSMZ	ACC-104, RRID:CVCL 2106

Supplementary Table 2. Clinical information on myeloid neoplasm patient samples

UPN	Clinical diagnosis	Sex	*JAK2* mutational status (VAF)	Gene mutational status
1	CML, blast phase	Male	Wild type	*CCND3* (G8D), *MAP3K14* (L728P)
2	AML with mutated *NPM1*	Female	Wild type	*CBL* (R585H), *DNMT3A* (F414S), *IDH1* (R132H), *NF1* (T862S), *EZH2* (S84*), *NPM1* (W288Cfs*12)
3	AML not other specified	Male	Wild type	*DNMT3A* (I715Hfs*19), *TET2* (Y1148Ifs*4)
4	ET	Female	V617F mutation (6%)	-
5	PMF	Male	V617F mutation (36%)	*TP53* (R248Q), *U2AF1* (Q157P), *ZRSR2* (R169*)
6	PV	Female	V617F mutation (95%)	-

Abbreviations: UPN, unique patient number; VAF, variant allele frequency; CML, chronic myeloid leukemia; AML, acute myeloid leukemia; ET, essential thrombocythemia; PMF, primary myelofibrosis; PV, polycythemia vera.

Supplementary Table 3. Human CARM1 shRNAs

shRNA for CARM1	Source	Identifier
Human CARM1-KD #1: TTTGTGTAAAATATGGCAGCGA	RNAi Core MSKCC	N/A
Human CARM1-KD #2: TTCAGGTACTTCTTGGCGTGGA	RNAi Core MSKCC	N/A
Human CARM1-KD #3: TAAAGGAATCAGGTTGTTGTGG	RNAi Core MSKCC	N/A

Supplementary Table 4. Human sgRNAs

sgRNA	Source	Identifier
Human CARM1-Y334F knock-in: TTCTGCCGTGCAGTACTTCC	Synthego	N/A
Human CARM1-Y149F knock-in: CCGCGGTGGATGAGTATTTC	Synthego	N/A
Human JAK1 knockout: CATCTTGTCATCAACGGTGA	Synthego	N/A
Human TYK2 knockout: TTGGGCCTGAGCATCGAAGA	Synthego	N/A

Supplementary Table 5. Recombinant peptides and proteins

Proteins	Source	Identifier
JAK2	Abcam	ab42619
Histone H3.1	New England Biolabs	M2503S
PAK1	Abcam	ab177574
CARM1	Reaction Biology	HMT-11-120
PRMT5	Reaction Biology	HMT-21-172
RUNX1b	MyBioSource	MBS1471613

Supplementary Table 6. Antibodies for immunoblotting

Antibodies	Source	Identifier	Dilution rate
Phosphotyrosine (clone 4G10)	Millipore Sigma	05-321X	1:1,000
Glutathione-S-Transferase (GST)	Sigma-Aldrich	G7781	1:2,000
CARM1	Bethyl Laboratories	A300-420A	1:1,000
BAF155	Santa Cruz Biotechnology	sc-365543	1:1,000
Asymmetric-dimethyl BAF155 Arg1064	Millipore	ABE1339	1:1,000
PABP1 (clone 10E10)	Abcam	ab6125	1:1,000
Asymmetric-dimethyl PABP1 Arg455/Arg460	Cell Signaling Technology	3505S	1:1,000
β-ACTIN (clone N-21)	Santa Cruz Biotechnology	sc-130656	1:15,000
JAK2 (mouse monoclonal) (clone 8E10.2)	EMD Millipore	04-001	1:1,000
JAK2 (rabbit polyclonal)	Cell Signaling Technology	3230S	1:1,000
Phosphorylated JAK2 Tyr1007/Tyr1008	Cell Signaling Technology	3771S	1:1,000
STAT5	Cell Signaling Technology	4807S	1:1,000
Phosphorylated STAT5 Tyr694	Cell Signaling Technology	9359S	1:1,000
ERK1/2	Cell Signaling Technology	4695S	1:1,000
Phosphorylated ERK1/2 Thr202/Tyr204	Cell Signaling Technology	9101S	1:1,000
AKT	Santa Cruz Biotechnology	sc-81434	1:1,000
Phosphorylated AKT Ser473	Cell Signaling Technology	4058S	1:1,000
JAK1	EMD Millipore	06-272	1:1,000
JAK3	Cell Signaling Technology	3775S	1:1,000
TYK2	Cell Signaling Technology	9312S	1:1,000
Histone 3	Abcam	ab24834	1:1,000
HSP90	ORIGENE	TA500494	1:2,000
SP1	Cell Signaling Technology	5931S	1:1,000
RUNX1 (mouse monoclonal)	Santa Cruz Biotechnology	sc-365644	1:500
RUNX1 (rabbit polyclonal)	Cell Signaling Technology	8229S	1:1,000
HA Epitope Tag Antibody	Rockland	600-445-384	1:1,000
MYC Epitope Tag Antibody	Rockland	600-445-381	1:1,000
Streptavidin	LI-COR	926-32230	1:1,000

Supplementary Table 7. TaqMan primers in quantitative RT-PCR

Primer	Source	Identifier
CARM1	Thermo Fisher Scientific	Hs00406354_m1
HPRT1	Thermo Fisher Scientific	Hs01003267_m1
BMI-1	Thermo Fisher Scientific	Hs00995536_m1
CD34	Thermo Fisher Scientific	Hs00990732_m1
ID2	Thermo Fisher Scientific	Hs0074379_m1
MIR144	Thermo Fisher Scientific	Hs04231506_s1
SATB1	Thermo Fisher Scientific	Hs00962580_m1
BCL2	Thermo Fisher Scientific	Hs01048932_g1
BCL2A1	Thermo Fisher Scientific	Hs06637395_s1
SMAD3	Thermo Fisher Scientific	Hs00969210_m1
CCND2	Thermo Fisher Scientific	Hs00153380_m1
KIF5B	Thermo Fisher Scientific	Hs01037194_m1
MIR223	Thermo Fisher Scientific	Hs04333910_s1

Supplementary Table 8. Chemicals

Proteins	Source	Identifier
Ruxolitinib	Cayman Chemical Company	11609
EPZ025654	Epizyme	N/A
CPZ868	MedChemExpress	HY-18960

3. Supplementary reference

1. Matsumoto N, Mori S, Hasegawa H, Sasaki D, Mori H, Tsuruda K, et al. Simultaneous screening for JAK2 and calreticulin gene mutations in myeloproliferative neoplasms with high resolution melting. Clinica Chimica Acta. 2016;462:166-173.
