# Supplementary material for: Tyrosine phosphorylation of CARM1 promotes its enzymatic activity and alters its target specificity
Source: Nat Commun. 2024 Apr 22;15:3415. doi: 10.1038/s41467-024-47689-4 (PMC11035800; doi:10.1038/s41467-024-47689-4)
Supplement: Supplementary file 3 — Description of Additional Supplementary Files [file 41467_2024_47689_MOESM3_ESM.docx]

**Description of Additional Supplementary Files**

**Supplementary Data 1:**

Significant proteins interacting with CARM1 in HEL cells using BioID system

Contaminant Repository for Affinity Purification (CRAPome, https://www.crapome.org/) were used as statistical tools to determine a comprehensive characterization of background contamination and identify specific high-confidence interactions from the data of our mass spectrometry. A cutoff frequency of ≥80% was applied in CRAPome database except that the average spectral count fold change ≥3.0 was assigned to high-confidence interactions.

**Supplementary Data 2:**

Significant proteins interacting with CARM1 in K562 cells using BioID system
